# Ethnic and trans-ethnic genome-wide association studies identify new loci influencing Japanese Alzheimer’s disease risk

**DOI:** 10.1038/s41398-021-01272-3

**Published:** 2021-03-03

**Authors:** Daichi Shigemizu, Risa Mitsumori, Shintaro Akiyama, Akinori Miyashita, Takashi Morizono, Sayuri Higaki, Yuya Asanomi, Norikazu Hara, Gen Tamiya, Kengo Kinoshita, Takeshi Ikeuchi, Shumpei Niida, Kouichi Ozaki

**Affiliations:** 1 Medical Genome Center, National Center for Geriatrics and Gerontology, Obu, Aichi 474-8511 Japan; 2 Department of Medical Science Mathematics, Medical Research Institute, Tokyo Medical and Dental University (TMDU), Tokyo, 113-8510 Japan; 3 RIKEN Center for Integrative Medical Sciences, Yokohama, Kanagawa 230-0045 Japan; 4 Department of Molecular Genetics, Brain Research Institute, Niigata University, Niigata, 951-8585 Japan; 5 Tohoku Medical Megabank Organization, Tohoku University, Sendai, Miyagi 980-8573 Japan; 6 Statistical Genetics Team, RIKEN Center for Advanced Intelligence Project, Tokyo, 103-0027 Japan; 7 Graduate School of Information Sciences, Tohoku University, Sendai, Miyagi 980-8579 Japan

**Keywords:** Medical genetics, Molecular neuroscience

## Abstract

Alzheimer’s disease (AD) has no cure, but early detection and risk prediction could allow earlier intervention. Genetic risk factors may differ between ethnic populations. To discover novel susceptibility loci of AD in the Japanese population, we conducted a genome-wide association study (GWAS) with 3962 AD cases and 4074 controls. Out of 4,852,957 genetic markers that passed stringent quality control filters, 134 in nine loci, including *APOE* and *SORL1*, were convincingly associated with AD. Lead SNPs located in seven novel loci were genotyped in an independent Japanese AD case–control cohort. The novel locus *FAM47E* reached genome-wide significance in a meta-analysis of association results. This is the first report associating the *FAM47E* locus with AD in the Japanese population. A trans-ethnic meta-analysis combining the results of the Japanese data sets with summary statistics from stage 1 data of the International Genomics of Alzheimer’s Project identified an additional novel susceptibility locus in *OR2B2*. Our data highlight the importance of performing GWAS in non-European populations.

## Introduction

The number of people with dementia is rapidly increasing and is estimated that it will reach 135 million worldwide by 2050^1^. Alzheimer’s disease (AD) is the most common cause of dementia among the elderly and the most frequent multifactorial neurodegenerative disease^2,3^. To date, there are no curative treatments for patients who already have AD, and available treatments are only able to delay the progression of the disease^4^. Thus, the disease has become a major global public health issue.

The majority of AD cases are sporadic and diagnosed in people over 65 years of age (late-onset AD: LOAD). LOAD is a heterogeneous disorder with complex interactions between genetic and environmental risk factors, and it is influenced by multiple common variants with low effect sizes^5,6^. Estimates of genetic heritability range between 60 and 80%^7^. A large number of genetic factors contribute to the etiopathogenesis and progression of AD. Amyloid precursor protein (*APP*), presenilin 1 (*PSEN1*), and presenilin 2 (*PSEN2*) have been identified as causes of autosomal-dominant AD^8^. The ε4 polymorphism in the protein encoded by the apolipoprotein E (*APOE*) gene, located on chromosome 19, is considered to be the strongest genetic risk factor for LOAD^9^. However, the *APOE ε*4 effect only accounts for 27.3% of the overall heritability^10^, and a large proportion of the heritability remains unexplained.

Many attempts have been made to identify additional genetic risk factors. The development of high-throughput genotyping and massively parallel next-generation sequencing has enabled genome-wide association studies (GWAS), which have successfully identified several genetic risk factors that affect the development of AD^5,6,11,12^. These findings are expected not only to explain some of the heritability of AD, but also to contribute to the understanding of the underlying etiologic, biologic, and pathologic mechanisms of AD.

Trans-ethnic meta-analyses of GWAS data improve the power to detect more common risk variants by increasing the sample size. Many susceptibility loci for AD have been reported, especially for American and European populations^6,13^. Some ethnicity-specific genetic risk factors have also been reported. Simino et al.^14^ reported ethnicity-specific genes associated with differences in plasma amyloid-β (Aβ) concentrations between African Americans and European Americans. We also previously reported an ethnicity-specific (Japanese) rare variant in *SHRAPIN* that is associated with increased risk of AD^15^. These findings should be addressed in further investigation using ethnicity-specific data sets with larger sample sizes, but there are few reports of large-scale GWAS data for AD in a Japanese population^16,17^.

Here, we comprehensively examined the genetic architecture of AD based on Japanese GWAS data obtained from a large sample. We discovered *FAM47E*, a novel ethnicity-specific locus with a significant genome-wide association for AD in the Japanese population. We also found six significant quantitative trait loci (QTLs) correlated with *FAM47E* SNP genotypes, as well as two AD susceptibility candidates (*RECK* and *TIMP3*) through gene-set analysis. Furthermore, we identified an additional novel AD susceptibility locus (*OR2B2*) through a trans-ethnic meta-analysis. Our data highlight the importance of performing GWAS in ethnicity-specific population data.

## Materials and methods

### Subjects

The discovery stage of the study consisted of 3962 AD cases and 4074 controls (2974 AD cases and 3096 controls from the National Center for Geriatrics and Gerontology [NCGG biobank]; 988 AD cases and 978 controls from Niigata University^16^). The control subjects with normal cognitive function that had subjective cognitive complaints, but normal cognition on the neuropsychological assessment, were categorized as normal controls. The case subjects were diagnosed with a probable or possible AD based on the criteria of the National Institute on Aging Alzheimer’s Association workgroups^18,19^. The average age was 74.6 years (standard deviation [SD] = 7.5 years) and the female-to-mate ratio was 1.51:1. The independent replication samples were composed of 1216 AD cases and 2446 controls (530 AD cases and 2446 controls from the NCGG biobank, 686 AD cases from the BioBank Japan Project^20,21^). The average age was 75.5 years (SD = 6.5 years) and the female-to-male ratio was 1.37:1. Genomic DNA was extracted from peripheral blood leukocytes by standard protocols using a Maxwell RSC Instrument (Maxwell RSC Buffy Coat DNA Kit, Promega, USA). All subjects were of Japanese origin and provided informed consent in writing. This study was approved by the ethics committee of each institution.

### Genotyping and quality control in the GWAS

Genome-wide genotyping in the discovery stage was performed by using the Affymetrix Japonica Array^22^ for the NCGG subjects and Affymetix GeneChip 6.0 microarrays for Niigata subjects^16^. Genotype imputation was conducted by using IMPUTE2^23^ with the 3.5 K Japanese reference panel developed by Tohoku Medical Megabank Organization (https://www.megabank.tohoku.ac.jp/english/) for NCGG subjects and the 1000 Genomes Project reference panel (1000 Genomes Phase 3^24^) for Niigata subjects. We used imputed variants with an INFO score ≥0.4 in the association analysis. Quality control (QC) was performed in each dataset separately after imputation using PLINK software^25^. We first applied QC filters to the subjects: (1) sex inconsistencies (--check-sex), (2) inbreeding coefficient (--het 0.1), (3) genotype missingness (--missing 0.05), (4) kinship coefficient (--genome 0.2), and (5) exclusion of outliers from the clusters of East Asian populations in a principal component analysis that was conducted together with 1000 Genomes Phase 3 data. We next applied QC filters to the genetic markers (SNPs and Indels): (1) genotyping efficiency or call rate (--geno 0.95), (2) minor allele frequency (--freq 0.001), and (3) Hardy–Weinberg equilibrium (--hwe 0.001). The common autosomal variants with the same effect alleles that passed these QC criteria were assessed with a logistic regression model, adjusting for sex and age with PLINK software (--logistic)^25^. We also conducted a meta-analysis combining the GWAS summary statistics of the NCGG and Niigata GWAS data for loci detected in the logistic regression model, which was implemented in METASOFT^26^. The Meta-*P* values were calculated based on Han and Eskin’s modified random effects model (RE-HE), which is optimized to detect associations under effect heterogeneity^26^.

### Replication study

Of the loci that satisfied *P* < 5.0 × 10^−6^ in the GWAS, lead markers were genotyped in the replication study. SNP genotyping was performed with the multiplex PCR-based Invader assay (Third Wave Technologies, Madison, WI). Association analysis in the replication study was performed by using a logistic regression model adjusted for sex and age with PLINK software (--logistic)^25^. The combined analysis of the GWAS and replication study was verified with the logistic regression method. To examine if the lead SNPs located on the association signals had independent effects, we further performed conditional logistic regression analyses on each lead SNP using association signals of the locus with *P* < 1.0 × 10^−4^. Association results were visualized with Q-Q and Manhattan plots created with the R package *qqman*. Functional motifs around the association signals were also investigated using the HaploReg V4.1 database^27^ (https://pubs.broadinstitute.org/mammals/haploreg/haploreg.php), including data from the Encyclopedia of DNA Elements (ENCODE)^28^ and the Roadmap Epigenomics projects^29^.

### Functional annotation

Functional annotation of the GWAS results, including that of genes mapping to the identified risk loci, was conducted using the FUMA web application (https://fuma.ctglab.nl/)^30^. FUMA requires GWAS summary statistics. Independent significant SNPs in the GWAS summary statistics were identified based on their *P* values (*P* < 5.0 × 10^−6^) and independence from each other (*r*^2^ < 0.6 in the 1000 Genomes phase 3 ALL of the reference panel population^24^) within a 250-kb window. Gene-set and tissue-expression analyses were performed with MAGMA implemented by FUMA^30^. The MAGMA gene-set analysis assessed over-representation of biological functions based on gene annotations using curated gene sets and gene ontology (GO) terms obtained from the Molecular Signature Database (MsigDB v5.2)^31,32^. Gene sets with Bonferroni-corrected *P*_bon_ < 0.05 were considered to be significantly enriched. The MAGMA tissue-expression analysis was conducted with eQTL data from the Genotype-Tissue Expression project (GTEx v8)^33^ to identify the tissue specificity of the phenotype.

### Genetic correlations

To estimate the SNP heritability of complex traits and diseases and to estimate the genetic correlation between different phenotypes, we implemented LD Hub^34^, an online platform for performing LD score regression using GWAS summary statistics data, available at http://ldsc.broadinstitute.org/.

### Trans-ethnic meta-analysis

For the trans-ethnic meta-analysis, we used two sets of the ethnic-specific GWAS summary statistics: our Japanese GWAS and the IGAP stage 1 data (21982 AD cases and 41944 controls)^6^. This trans-ethnic meta-analysis was implemented in METASOFT^26^. The Meta-*P* values were calculated based on Han and Eskin’s modified random effects model (RE-HE). Of the loci that satisfied Meta-*P* (RE-HE) < 5.0 × 10^−6^ in the trans-ethnic meta-analysis, lead markers were genotyped using the independent replication samples. The combined analysis of our Japanese GWAS and replication study was verified by conducting logistic regression with adjustment for sex and age using PLINK software (--logistic)^25^.

### Quantitative trait locus analysis

We obtained 60 quantitative traits from our NCGG routine blood tests. To examine the relationships between the hematological traits and SNP genotypes, QTL analysis was conducted using linear regression analysis with adjustment for sex and age implemented by PLINK software (--linear)^25^.

## Results

### GWAS in the Japanese population

**Fig. 1:**
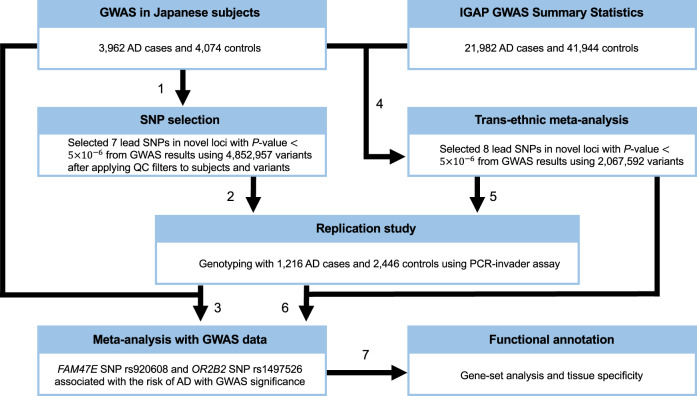
This study is performed based on the following 7 steps. Workflow of the study.

**Fig. 2:**
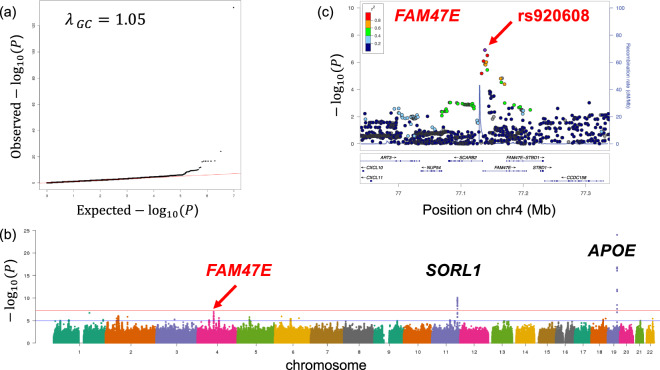
Genome-wide association study (GWAS) of AD in the Japanese population. **a** Quantile-quantile plots of the genome-wide *P* values, **b** A Manhattan plot of the GWAS, and **c** Regional association plot of the variants in the *FAM47E* locus.

We examined association signals from a Japanese GWAS data set of 3962 AD cases and 4074 controls with genotype imputation (Fig. 1). A total of 4,852,957 genetic markers (single-nucleotide polymorphisms [SNPs] and short insertions and deletions [Indels]) passed stringent QC filters for both genotypes and samples after SNP imputation. The associations were assessed with logistic regression, adjusting for sex and age (see Materials and methods section). A quantile–quantile plot of the genome-wide *P* values indicated no genomic inflation (*λ*_GC_ = 1.05, Fig. 2a). A total of 134 genetic markers, located within nine genes (*APOE*, *SORL1*, *FAM47E, PAPOLG, RAB3C, BANK1, LINC01867, LINC00899*, and *LOC101928561*) showed suggestive associations (*P* < 5.0$\times 10^{ - 6}$, Fig. 2b). The *APOE* and *SORL1* genes have been reported as AD susceptibility loci in several populations^6,11,16^ and the remaining seven loci (Supplementary Fig. 1) have not previously been associated with AD in the Japanese population. We also carried out an ethnic meta-analysis combining the GWAS summary statistics of our NCGG and Niigata data on the seven loci using METASOFT. The logistic regression and ethnic meta-analysis showed similar *P* values for AD associations (Table 1).

**Table 1:** Summary statistics of newly identified regions significantly associated with Alzheimer’s disease.

SNP	Allele 1/2	Closest gene	Stage (*n*)	No. of subjects		Allele 1 frequency		OR	95% CI	*P* (Meta-*P*)
				Case	Control	Case	Control			
**rs920608**	**C/A**	***FAM47E***	**GWAS**^**a**^ **(8030)**	**3959**	**4071**	**0.030**	**0.046**	**0.63**	**0.53**–**0.75**	**1.25** **×** **10**^**−7**^ **(6.09** **×** **10**^**−8**^**)**
			**Replication**^**a**^ **(3662)**	**1216**	**2446**	**0.037**	**0.046**	**0.74**	**0.57**–**0.97**	**2.72** **×** **10**^**−2**^
			**Combined**^**a**^ **(11692)**	**5175**	**6517**	**0.032**	**0.046**	**0.65**	**0.57**–**0.75**	**5.34** **×** **10**^**−9**^
rs13386821	T/G	*PAPOLG*	GWAS^a^ (8030)	3958	4072	0.13	0.16	0.79	0.72–0.87	1.10 × 10^−6^ (7.08 × 10^−5^)
			Replication^a^ (3652)	1144	2508	0.15	0.14	1.05	0.91–1.22	0.50
			Combined^a^ (11682)	5102	6580	0.13	0.15	0.86	0.79–0.92	8.54 × 10^−5^
rs35734784	T/C	*RAB3C*	GWAS^a^ (7959)	3921	4038	0.32	0.36	0.85	0.79–0.91	1.70 × 10^−6^ (1.75 × 10^−6^)
			Replication^a^ (3497)	1084	2413	0.35	0.34	1.03	0.93–1.16	0.55
			Combined^a^ (11456)	5005	6451	0.33	0.35	0.90	0.85–0.95	2.09 × 10^−4^
rs17249850	T/C	*BANK1*	GWAS^a^ (8035)	3961	4074	0.0092	0.017	0.49	0.37–0.66	2.76 × 10^−6^ (8.26 × 10^−6^)
			Replication^a^ (3660)	1148	2512	0.0091	0.014	0.64	0.38–1.08	0.097
			Combined^a^ (11695)	5109	6586	0.0092	0.016	0.55	0.42–0.71	3.51 × 10^−6^
rs2727868	C/T	*LINC01867*	GWAS^a^ (8001)	3943	4058	0.35	0.38	0.85	0.80–0.91	3.29 × 10^−6^ (7.30 × 10^−6^)
			Replication^a^ (3644)	1141	2503	0.36	0.36	1.02	0.91–1.14	0.72
			Combined^a^ (11645)	5084	6561	0.36	0.37	0.91	0.86–0.96	5.07 × 10^−4^
rs4605844	A/G	*LOC101928516*	GWAS^a^ (8004)	3946	4058	0.43	0.39	1.17	1.09–1.25	3.99 × 10^−6^ (4.03 × 10^−6^)
			Replication^a^ (3649)	1143	2506	0.41	0.43	0.94	0.84–1.04	0.23
			Combined^a^ (11653)	5089	6564	0.42	0.41	1.09	1.03–1.15	3.45 × 10^−3^
rs4073601^b^	G/A	*LINC00899*	GWAS^a^ (7863)	3876	3987	0.12	0.14	0.80	0.73–0.89	1.90 × 10^−5^ (4.21 × 10^−5^)
			Replication^a^ (3501)	1088	2413	0.13	0.14	0.90	0.77–1.05	0.19
			Combined^a^ (11364)	4964	6400	0.12	0.14	0.83	0.76–0.90	6.88 × 10^−6^

*GWAS* genome-wide association study, *OR* odds ratio.

^a^*P* values and ORs are adjusted for age and gender by logistic regression analysis under an additive model.

^b^The rs4073601 SNP was used as a proxy of the rs8137273 SNP (GWAS *P* = 3.80 × 10^−6^).

### Replication study and ethnicity-specific meta-analysis

The lead SNPs located on the seven novel loci (*FAM47E, PAPOLG, RAB3C, BANK1, LINC01867, LINC00899*, and *LOC101928561*, Supplementary Fig. 1) were genotyped using an independent Japanese AD case–control cohort of 1216 AD cases and 2446 controls (Table 1). As the lead SNP of *LINC00899* (rs8137273; GWAS *P* = 3.80 × 10^−6^) showed poor signals in the PCR-based Invader assay, a proxy SNP (rs4073601; GWAS *P* = 1.90 × 10^−5^) was used in the subsequent meta-analysis combining results from the GWAS and replication data sets. Of the seven lead SNPs, the *FAM47E* SNP showed modest evidence of association in the replication study (*P* < 0.05, Fig. 2c and Table 1), and the subsequent meta-analysis combining results from the GWAS and replication data sets reached genome-wide significance in a logistic regression (*n* = 11692, *P* = 5.34 × 10^−9^, odds ratio = 0.65, 95% CI = 0.57–0.75, Table 1). As the *FAM47E* SNP genotypes obtained from the GWAS data set were imputed, we genotyped all of samples in the GWAS data set (*n* = 7562) by PCR-invader assay and evaluated the concordance rate of the imputed SNPs, which provided genotypes of high concordance (0.9963), and obtained similar association for AD with statistical *P* value of 6.24 × 10^−7^. The subsequent meta-analysis combining results from the GWAS and replication data sets also reached genome-wide significance in a logistic regression (*n* = 11229, *P* = 1.72 × 10^−8^, odds ratio = 0.65, 95% CI = 0.56–0.76).

The most significant SNP of *FAM47E* was rs920608 (Table 1 and Fig. 2c). Because 11 genetic markers other than rs920608 were included in the locus with *P* < 1.0 × 10^−4^, we further examined whether these markers had independent effects using conditional logistic regression analysis on rs920608, but no independent association signals were found (Supplementary Table 1).

**Fig. 3:**
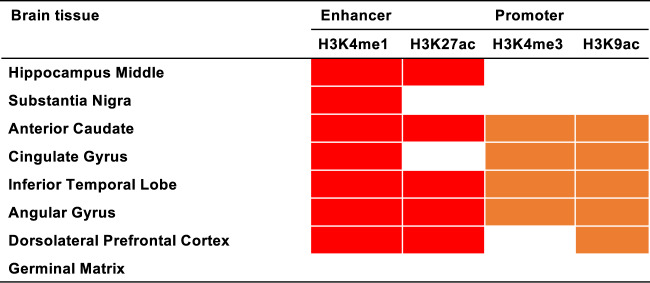
Histone marks associated with enhancers or promoters in several brain regions on rs920608. Enhancers and promoters in eight brain tissues.

The minor allele frequencies (MAFs) of rs920608 in different populations were African (AFR) = 0.11, Mixed American (AMR) = 0.016, European (EUR) = 0.0060, East Asian (EAS) = 0.038, and South Asian (SAS) = 0.021 in the 1000 Genomes Project Phase 3 data^24^, and AFR = 0.09, AMR = 0.013, EUR = 0.0073, and EAS = 0.043 in the Genome Aggregation Database (gnomAD)^35^. The MAF of the EAS population (including Japanese) was statistically significantly different from that of each of the other populations (*P* < 0.05 with Fisher’s exact test, Supplementary Table 2). Furthermore, the HaploReg database showed that this SNP overlaps histone marks associated with enhancers (H3K4me1 and H3K27ac) or promoters (H3K4me3 and H3K9ac) in several brain tissues^27^ (Fig. 3). These results indicate that the *FAM47E* locus has the potential to be an East Asian–specific AD susceptibility locus.

### Expression quantitative trait loci

We investigated the effect of novel AD variants on the expression of genes by assessing expression QTLs (eQTLs) using the Genotype-Tissue Expression Portal (GTEx; https://www.gtexportal.org/home/). The A allele for AD risk at rs920608, the top SNP for the *FAM47E* locus, was associated with increased expression of *SCARB2* in the adrenal gland (*P* = 0.007). The proxies for rs920608 also had eQTLs for *FAM47E* and *SCARB2* in several brain tissues (*P* < 0.05).

### Functional gene annotations and genetic correlations

**Fig. 4:**
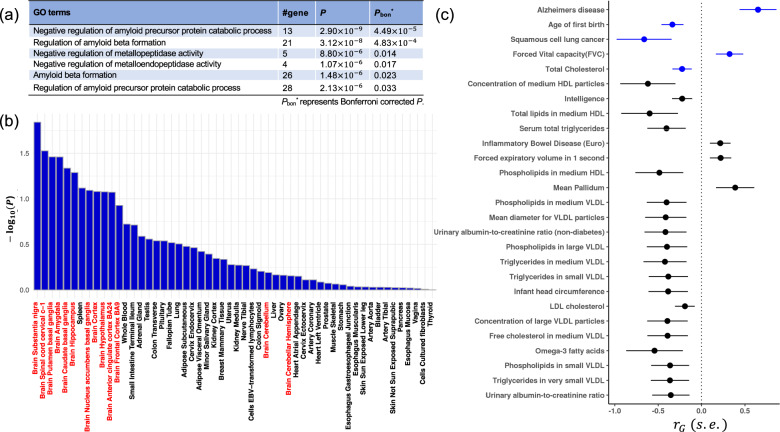
Gene-set analysis and genetic correlations. **a** *P*_bon_^*^ represents the Bonferroni-corrected *P*. **b** The red lettering indicates brain regions. **c** Diseases and traits with genetic correlations with AD (*P* < 0.1) are shown. Those shown with blue symbols represent diseases and traits with *P* < 0.05.

To gain biological insight from the functional annotation of the GWAS results, we performed gene-set analysis and tissue-expression analysis by using MAGMA^36^ implemented by FUMA^30^. The MAGMA gene-set analysis identified six GO terms with Bonferroni-corrected *P*_bon_ < 0.05 when applying our ethnicity-specific GWAS summary statistics (Fig. 4a). Of the six, four were associated with amyloid precursor proteins, known pathological hallmarks of AD, and the remaining two were novel proteins associated with negative regulation of metallopeptidase and metalloendopeptidase activities (Fig. 4a). These two GO terms shared four genes (*RECK*, *PICALM*, *SORL1*, and *TIMP3*), of which two (*RECK* and *TIMP3*) have never been reported as AD susceptibility loci. However, both are target genes of microRNA-21^37^, which has been reported to inhibit cell apoptosis induced by Aβ_1–42_ and which has a protective role in AD^38^. Moreover, we examined the tissue specificity of the phenotype with a MAGMA tissue-expression analysis, using RNA sequencing data of 54 tissue types obtained from GTEx v8^33^. Many of top-ranked tissue types observed were regions of the brain, although there were no tissue types significantly associated with AD (Fig. 4b).

To investigate the extent of genetic overlap between our Japanese GWAS data and phenotypes, we also estimated genetic correlations across the different diseases and traits available at LD Hub^34^ based on LD-score regression. Significant correlations were found for five traits. Two significant positive genetic correlations were observed: with AD in a European population (*r*_G_ = 0.65, *P* = 2.3 × 10^−3^) and with forced vital capacity (*r*_G_ = 0.32, *P* = 4.0 × 10^−2^). Three negative correlations were observed: with age of first birth (a woman’s age at the birth of her first child, *r*_G_ = −0.34, *P* = 8.1 × 10^−3^), squamous cell lung cancer (*r*_G_ = −0.66, *P* = 3.7 × 10^−2^), and total cholesterol (*r*_G_ = −0.22, *P* = 4.4 × 10^−2^; Fig. 4c). Although none were statistically significant after correction for multiple testing, these traits (age of first birth^11^, forced vital capacity^39^, squamous cell lung cancer^40^, and total cholesterol^41^) were reported in previous studies to correlate with AD. In addition, we estimated overall SNP heritability (${{h^{\mathrm{2}}}_{{\mathrm{SNP}}}}$) to be 0.16 (SE = 0.06) in the Japanese GWAS data, although Lee et al.^42^ estimated the ${{h^{\mathrm{2}}}_{{\mathrm{SNP}}}}$ to be 0.24 (SE = 0.03) in the European GWAS data.

### Trans-ethnic meta-analysis

To improve the power to detect additional AD susceptibility loci through a large number of samples, we performed a trans-ethnic meta-analysis combining the results of our Japanese GWAS data with the summary statistics from stage 1 of the International Genomics of Alzheimer’s Project (IGAP; 21982 AD cases and 41944 controls)^6^. The trans-ethnic meta-analysis was carried out on 2,067,592 genetic markers with Han and Eskin’s modified random effects model (RE-HE) implemented by METASOFT. Of these markers, 26 loci reached a Meta-*P* (RE-HE) <5.0 × 10^−6^, of which 18 were located on genes known to contribute to AD risk (*APOE*, *PICALM*, *BIN1*, *CLU*, *CR1*, *MS4A4A*, *SORL1*, *MADD*, *HLA-DRA*, *CD2AP*, *EPHA1*, *ADAMTS1*, *SLC24A4*, *LACTB2*, *ELL*, *FERMT2*, *ZCWPW1*, and *TSPOAP1*, Supplementary Fig. 2). The lead SNPs located on the remaining eight novel loci (*MTSS1L*, *CLEC3B*, *EFL1*, *FAM155A*, *NTM*, *OR2B2*, *C1S*, and *TSPAN14*, Supplementary Fig. 2) were genotyped in an independent AD case–control cohort of Japanese samples (Supplementary Table 3). Finally, a subsequent meta-analysis combining results from the GWAS data, replication data, and IGAP data reached genome-wide significance for *OR2B2* (rs1497526, Meta-*P* = 2.14 × 10^−8^, Table 2). As the lead SNP of *OR2B2* (rs1497525) showed poor signals in the PCR-based Invader assay, a proxy SNP (rs1497526) was used in the subsequent meta-analysis combining results from the GWAS and replication data sets, and IGAP data. The proxy SNP was identified by the LDproxy Tool (https://ldlink.nci.nih.gov/?tab=ldproxy, *R*-squared = 1 in Japanese). As this proxy SNP was imputed in the Japanese GWAS data set, we genotyped all of samples in the GWAS data (*n* = 7734) by PCR-invader assay and evaluated the concordance rate of the imputed SNPs, which provided genotypes of high concordance (0.9919).

**Table 2:** Summary statistics of regions identified in the trans-ethnic meta-analysis.

Gene	Trans-ethnic meta-analysis (i)		proxy SNPs	OR (IGAP, GWAS)	Replication study (ii)				OR	95% CI	*P*	Meta-analysis (i)+(ii)
	SNP	Meta-*P*			No. of subjects		MAF					Meta-*P*
					Case	Control	Case	Control				
*MTSS1L*	rs7195572	1.83 × 10^−7^	–	0.94, 0.89	1128	2419	0.29	0.30	0.94	0.83–1.05	0.27	9.25 × 10^−8^
*CLEC3B*	rs7618668	6.43 × 10^−7^	rs7626571	0.88, 0.93	1129	2421	0.062	0.057	1.11	0.89–1.39	0.36	5.17 × 10^−6^
*EFL1*	rs905450	9.31 × 10^−7^	–	0.93, 0.90	1089	2260	0.27	0.26	1.01	0.89–1.14	0.92	5.60 × 10^−6^
*FAM155A*	rs9520713	2.33 × 10^−6^	–	0.93, 0.87	1090	2300	0.058	0.061	1.01	0.80–1.27	0.97	3.66 × 10^−6^
*NTM*	rs9787911	2.59 × 10^−6^	–	0.94, 0.96	1091	2282	0.50	0.49	1.00	0.90–1.11	0.99	4.43 × 10^−6^
***OR2B2***	**rs1497525**	**2.96** **×** **10**^**−6**^	**rs1497526**	**1.12, 1.18**	**1156**	**2385**	**0.15**	**0.12**	**1.34**	**1.15**–**1.56**	**1.98** **×** **10**^**−4**^	**2.14** **×** **10**^**−8**^
*C1S*	rs7311672	3.32 × 10^−6^	–	0.92, 0.88	1091	2300	0.065	0.071	0.86	0.70–1.07	0.19	2.72 × 10^−6^
*TSPAN14*	rs10748526	4.60 × 10^−6^	–	0.93, 0.93	1095	2309	0.15	0.15	0.93	0.80–1.08	0.35	3.75 × 10^−6^

*MAF* minor allele frequency, *OR* odds ratio, *SNP* single-nucleotide polymorphism.

To examine whether *OR2B2* is an ethnicity-specific susceptibility locus like *FAM47E*, we checked the MAF of *OR2B2* SNP rs1497526 for several populations in the 1000 Genomes Project Phase 3 data^24^ (AFR = 0.29, AMR = 0.091, EUR = 0.046, EAS = 0.081, and SAS = 0.076) and gnomAD^35^ (AFR = 0.24, AMR = 0.076, EUR = 0.032, and EAS = 0.043) and assessed the statistical significance of differences between EAS and each of the other populations. Statistically significant differences were observed between EAS and EUR, between EAS and AFR, and between EAS and AMR (*P* < 0.05 with Fisher’s exact test, Supplementary Table 2). These results show that *OR2B2* could be a common susceptibility locus among some populations. There is no eQTL in the GTEx database at rs1497526.

### Quantitative trait locus analysis for *FAM47E* and *OR2B2*

We also investigated the relationship between each of the *FAM47E* and *OR2B2* SNP genotypes and the hematological traits. QTL analysis was carried out on 60 blood test results using linear regression analysis with adjustment for sex and age (Supplemental Table 4). Six traits showed suggestive levels of associations with the *FAM47E* SNP (*P* < 0.05; I-BIL: indirect bilirubin, D-BIL: direct bilirubin, MPV: mean platelet volume, CRE: creatine, eGFR: estimated glomerular filtration rate, and GLU: glucose) and two traits with the *OR2B2* SNP (*P* < 0.05; TP: total protein, and MPV), although none were statistically significant after correction for multiple testing.

## Discussion

Although trans-ethnic meta-analyses, along with greatly increased sample sizes, have contributed to the identification of many genetic risk factors for AD^5,6,11^, there are few reports investigating ethnicity-specific associations with AD, especially for the Japanese population^16^. We succeeded in identifying a novel ethnicity-specific AD susceptibility locus within the *FAM47E* gene on chromosome 4. The difference in the allele frequency of the genotyped variants among populations could have resulted in the identification of the locus. The MAF of the *FAM47E* SNP (rs920608) was relatively high in the EAS population compared with the AMR and EUR populations. This SNP has also been shown to overlap histone marks associated with enhancers (H3K4me1 and H3K27ac) or promoters (H3K4me3 and H3K9ac) in several brain tissues from the HaploReg database^27^. Furthermore, the lead SNP and proxies had eQTLs for *FAM47E* and *SCARB2* in several brain tissues. These results suggest that *FAM47E* and *SCARB2* are likely to be functionally related to AD, although we should verify this using a larger sample size of Japanese AD cases.

The *FAM47E* locus has been associated with Parkinson’s disease (PD)^43,44^. Although PD and AD have remarkably different clinical and pathological features, the two diseases are the most common neurodegenerative disorders and show considerable overlap in the development of neurodegeneration. Previous studies have reported some common genetic loci that increase both PD and AD risk. Gregório et al.^45^ reported that *APOE4*, a strong risk factor for AD, is also associated with cognitive decline in PD. Allen et al.^46^ reported that polymorphisms in the glutathione S-transferase omega gene are associated with risk and age at onset of AD and PD. Li et al.^47^ has reported a common genetic factor in the *NEDD9* gene associated with both AD and PD. Thus, the *FAM47E* locus could be an AD-and-PD–associated locus. However, the associations of the glutathione S-transferase omega and *NEDD9* are only shown in relatively small data sets and they should be confirmed in subsequent studies with a large sample size. Furthermore, we found correlations between *FAM47E* SNP genotypes and six quantitative traits of blood test results, many of which (direct/indirect BIL^48^, MPV^49^, CRE^50^, and GLU^51^) have been associated with dementias, including AD. These results also support the view that *FAM47E* is an AD-associated locus.

The functional annotation of the GWAS results can provide biological insight. We detected six statistically significant GO terms using MAGMA^36^. Four of these GO terms were associated with amyloid precursor proteins, which are known pathological hallmarks of AD. The remaining two were novel and shared four genes including *SORL1*^16^ and *PICALM*^52,53^ which are well established AD genes. These results were not surprising, but they strengthen the credibility of our GWAS findings. Also, while two (*RECK* and *TIMP3*) of the four genes have never been reported as AD susceptibility loci, these genes are common targets of microRNA-21^37^, which has been reported to inhibit cell apoptosis induced by Aβ_1–42_ and to have protective roles in AD^38^. These two genes could, therefore, be candidate AD susceptibility genes.

We also identified a novel locus, *OR2B2*, in a meta-analysis combining the first GWAS data, its replication data, and IGAP data. However, because *OR2B2* has never been reported to be associated with AD, and no histone marks associated with enhancers or promoters were enriched in the *OR2B2* SNP in several brain tissues, further investigation will be needed to examine whether it has any functional association with AD. We finally assigned all SNPs in the IGAP data to nearest genes, and checked if any *FAM47E* SNPs and *OR2B2* SNPs had AD associations. However, there were no statistically significant associations in the two genes (Supplementary Table 5). There are power limitations and SNP array-based GWAS limitations in the current analyses. Early genome-wide SNP arrays, used for the Niigata subjects in our analysis, were developed based on tag SNPs from reference panels of European populations. Linkage disequilibrium patterns are different among ethnic groups and these arrays provided poor coverage in our Asian population. On the other hand, an ethnic-specific array (the Japonica Array) was used for our NCGG subjects, which should enable a possibly large harvest of ethnic-specific disease signals. When combining the early genome-wide SNP arrays and new ethnic-specific SNP arrays, larger and uniform reference panels will be preferred to improve genotype imputation. In addition, GWAS based on SNP arrays cannot detect rare variants associated with diseases^54^, and it should be necessary to increase a sample size to increase the power for detecting association signals with small effect sizes. We will perform further investigations with larger sample sizes to further validate the effectiveness of our findings in near future.

## Conclusions

We conducted a Japanese genome-wide association study of AD with a large sample size and a trans-ethnic meta-analysis with Caucasian GWAS data, and identified novel susceptibility loci for AD. Further investigation using well-powered GWAS in specific ethnic groups would likely identify additional genetic risk factors associated with AD, which causes a great deal of suffering for patients and their families and is also a leading cause of death in many countries. We anticipate that the identification of the genetic architecture of AD susceptibility and related pathways will help us to understand the pathogenesis of LOAD. In turn, this will contribute to innovative medical and pharmaceutical approaches that advance the development of precision medicine for this common but serious disorder.

## Supplementary information

Supplemental Figure 1

Supplemental Figure 2

Supplemental Table 1

Supplemental Table 2

Supplemental Table 3

Supplemental Table 4

Supplemental Table 5

## Data Availability

We used the open source program languages R (version 3.4.1) and Ruby (version 2.4.0) to analyse the data and create the plots. Code is available upon request from the corresponding authors.
