# Supplementary figures and images for: Ethnic and trans-ethnic genome-wide association studies identify new loci influencing Japanese Alzheimer’s disease risk
Source: Transl Psychiatry. 2021 Mar 3;11:151. doi: 10.1038/s41398-021-01272-3 (PMC7925686; doi:10.1038/s41398-021-01272-3)

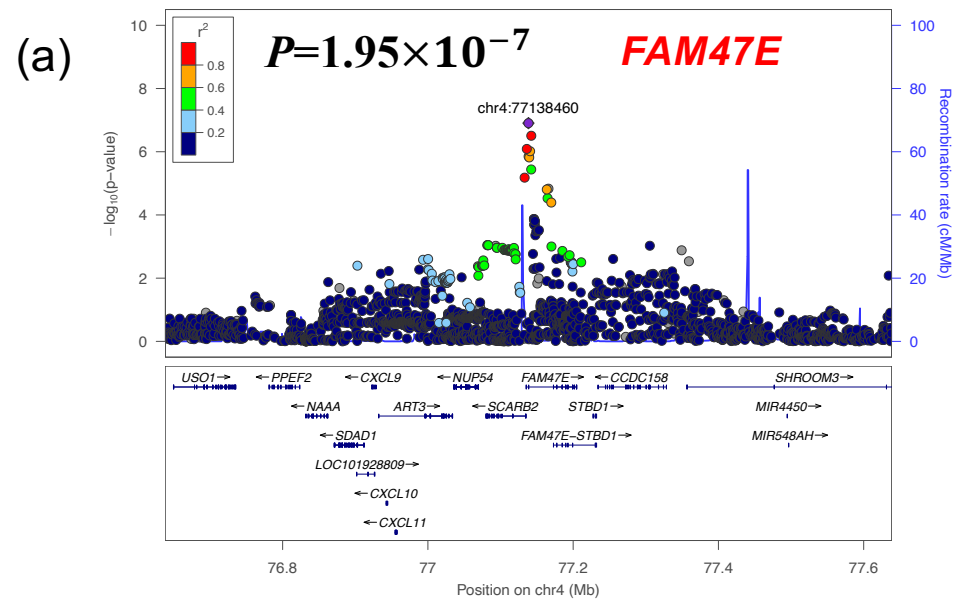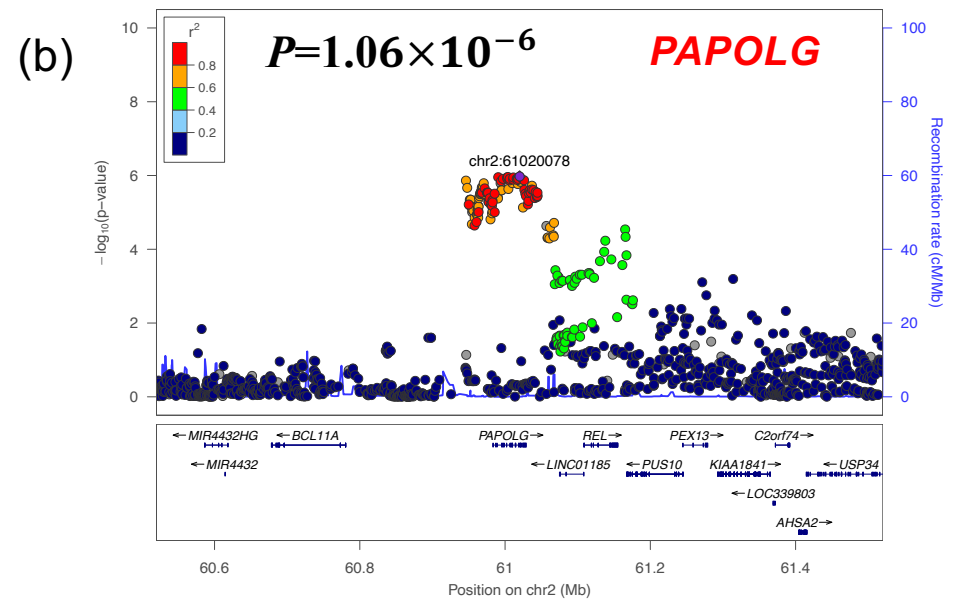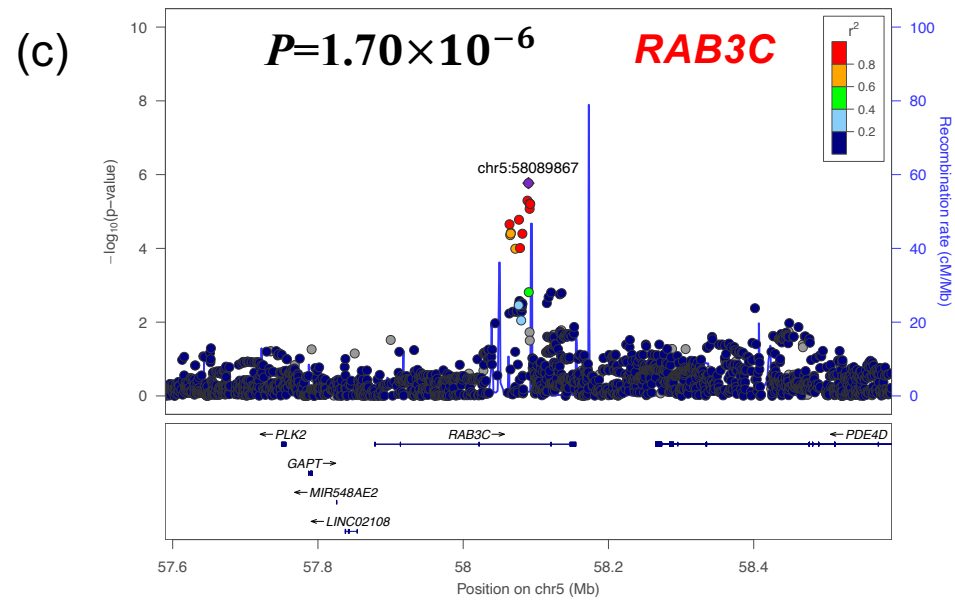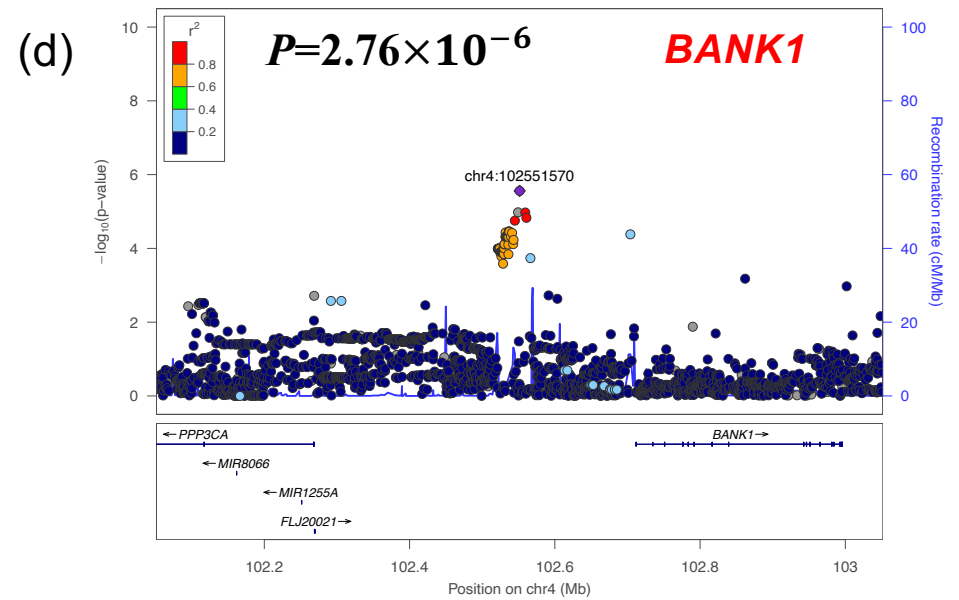

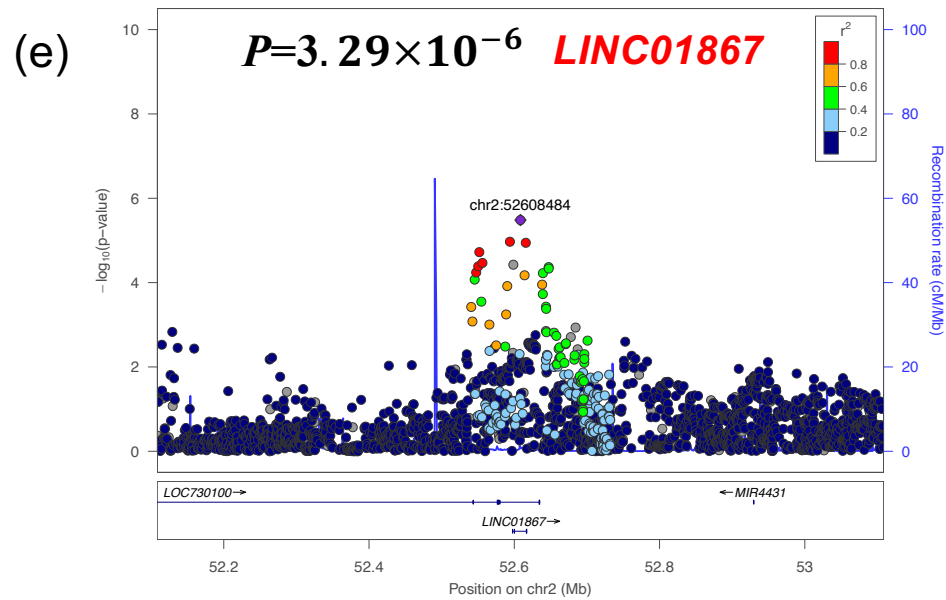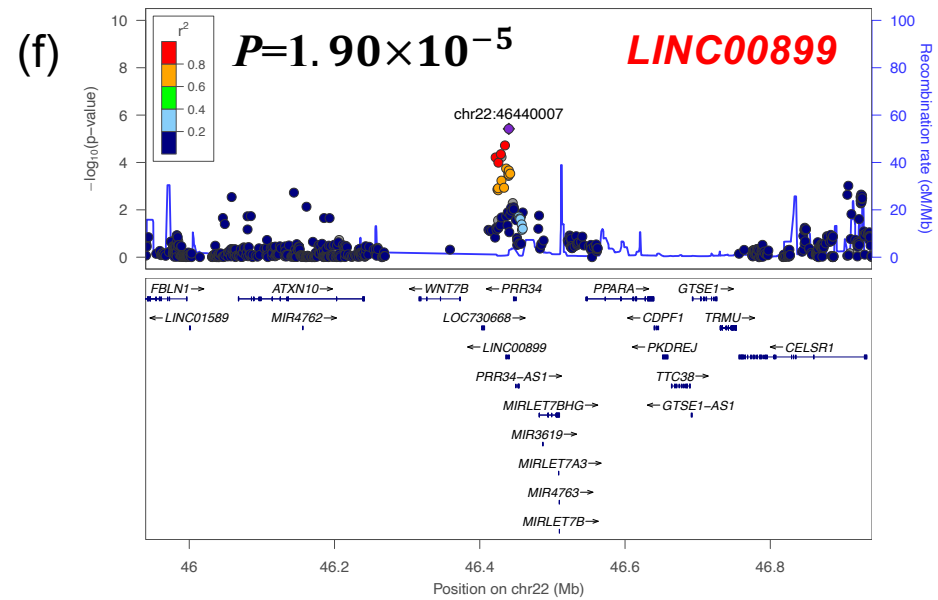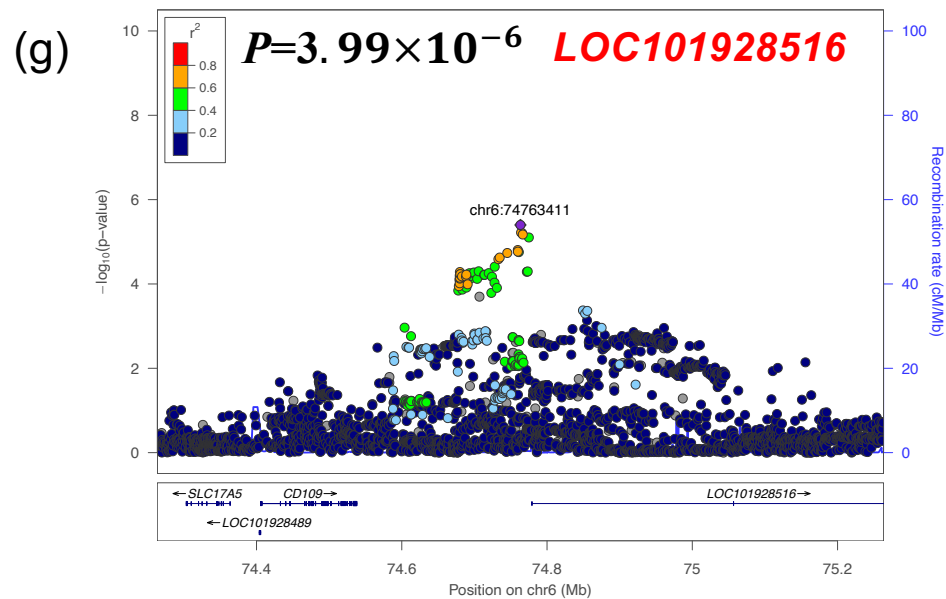

Supplement: Supplementary file 1 — Supplemental Figure 1 [file 41398_2021_1272_MOESM1_ESM.pdf]

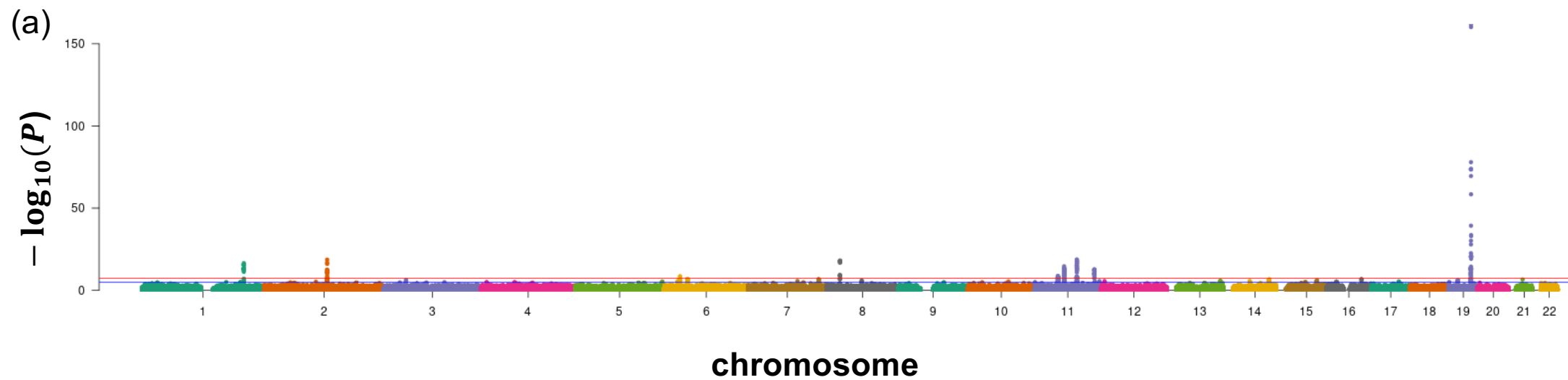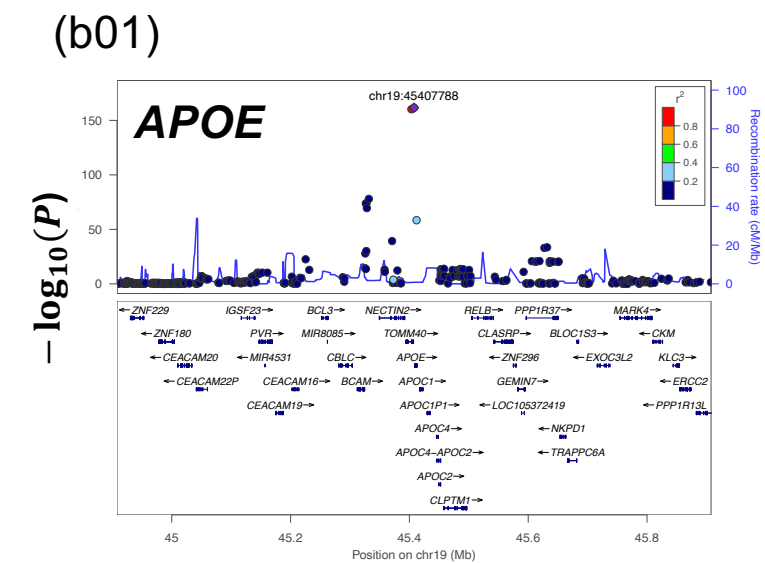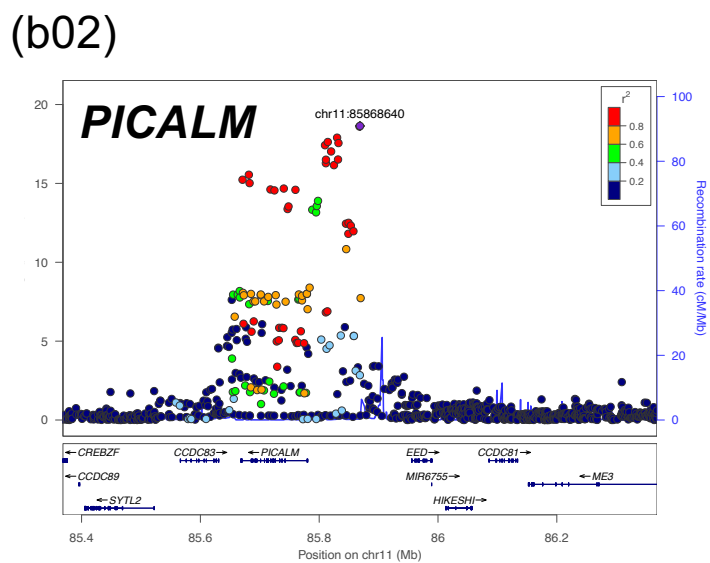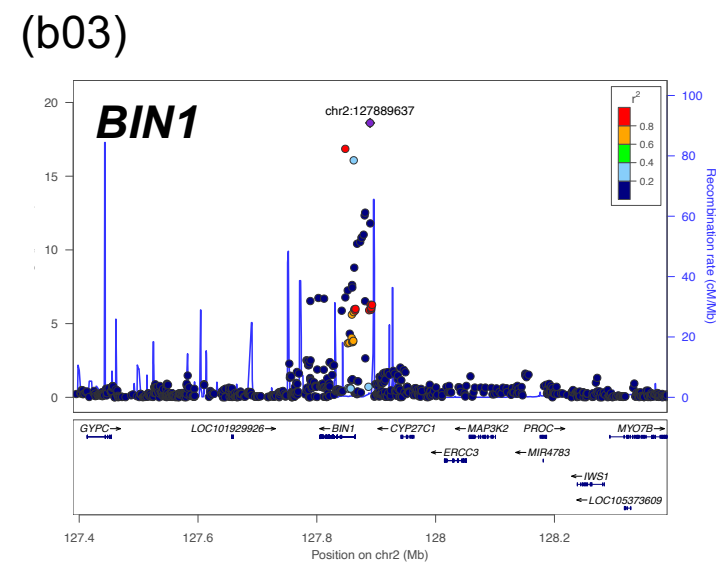

(b04)

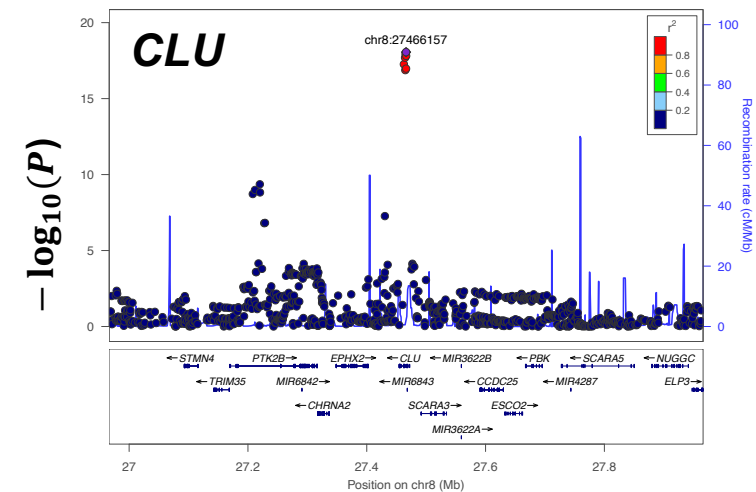

(b05)

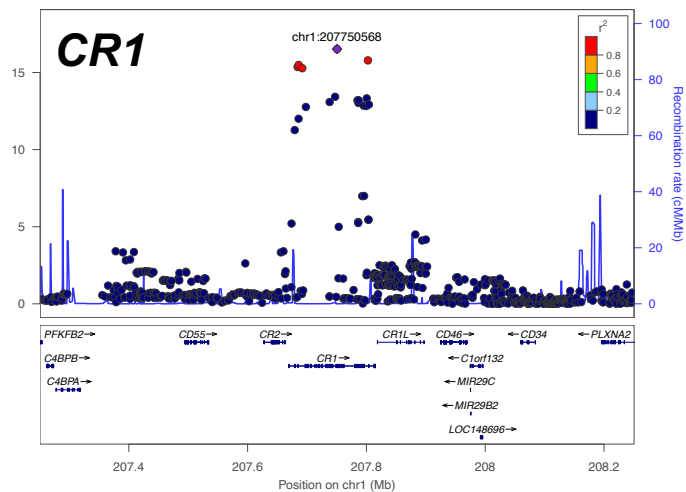

(b06)

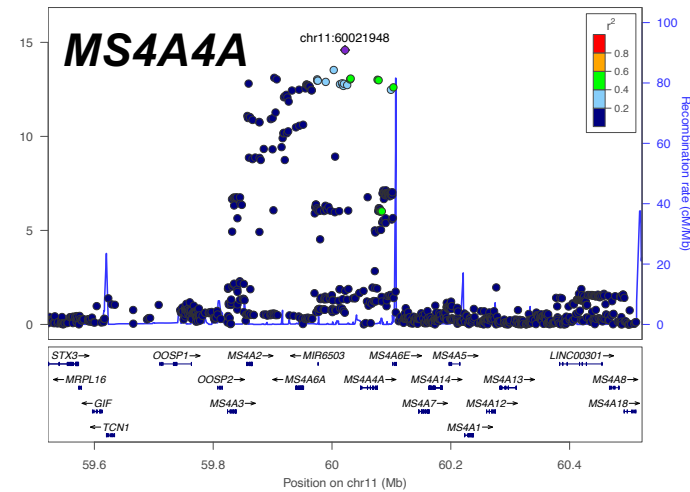

(b07)

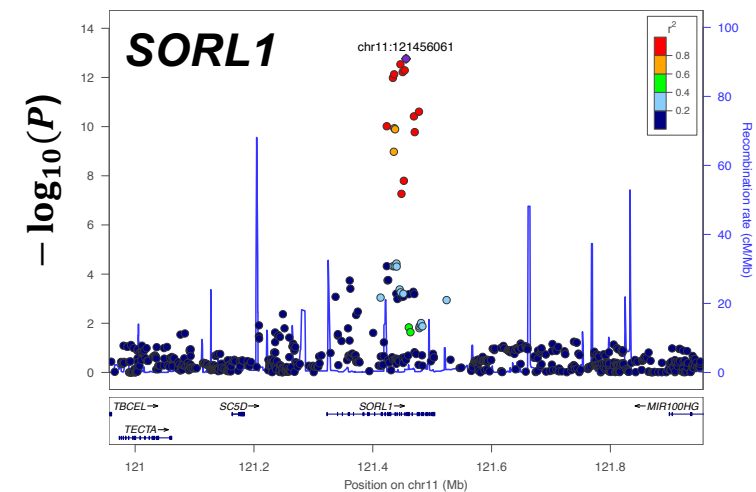

(b08)

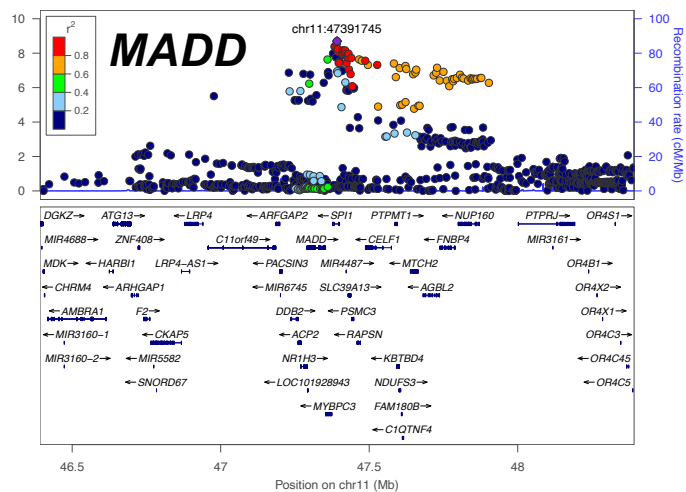

(b09)

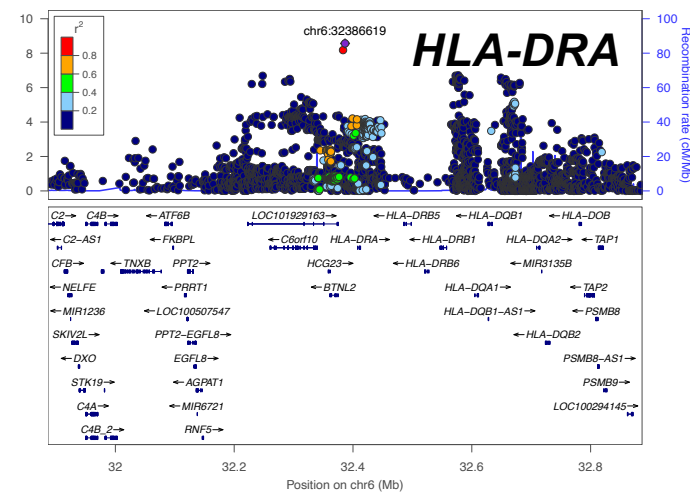

(b10)

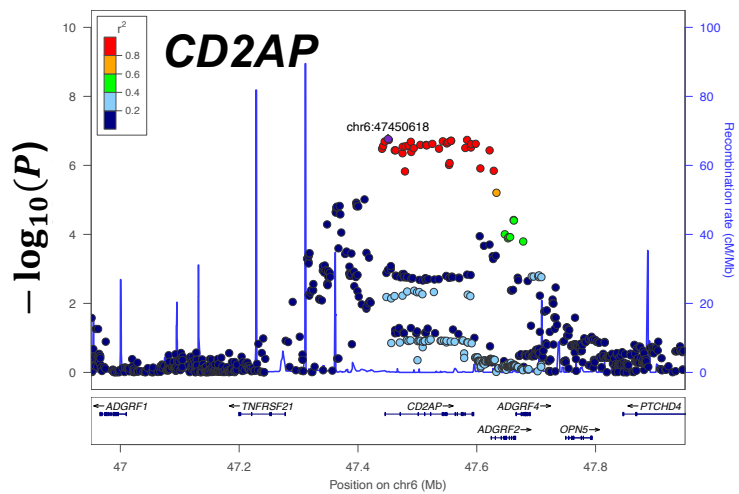

(b11)

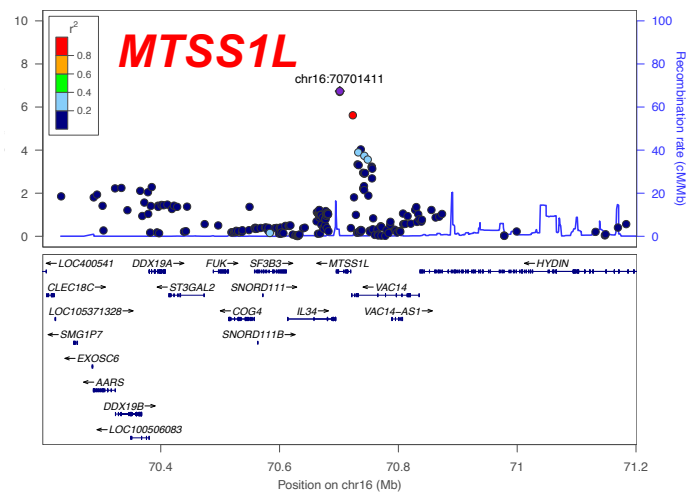

(b12)

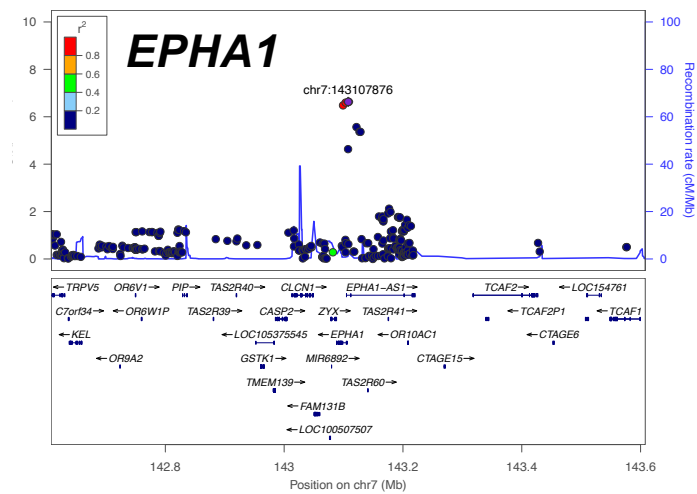

(b13)

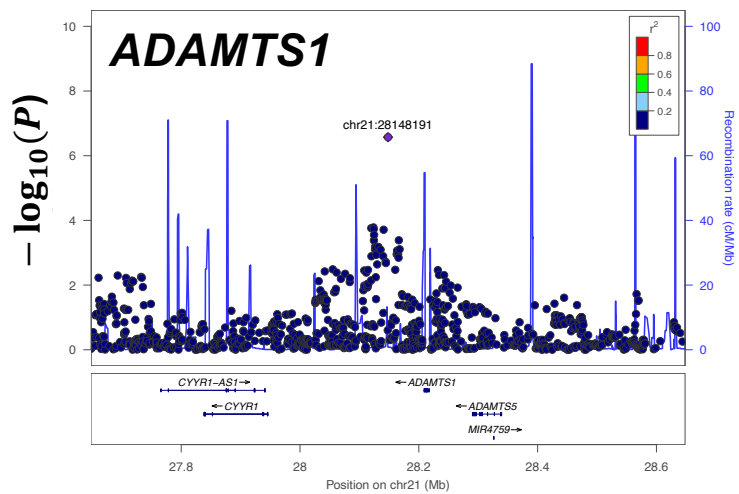

(b14)

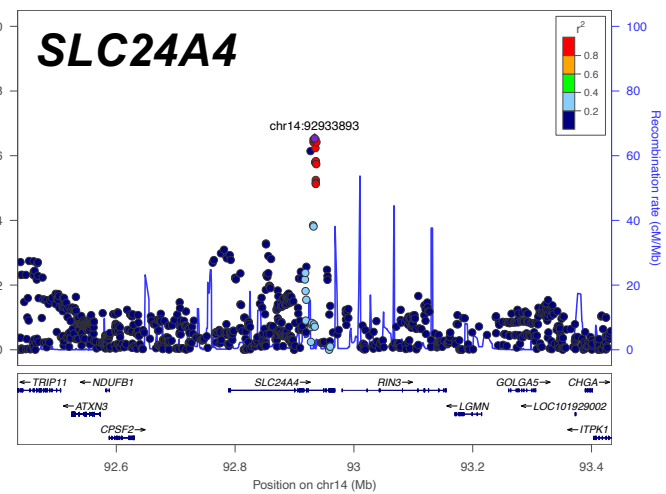

(b15)

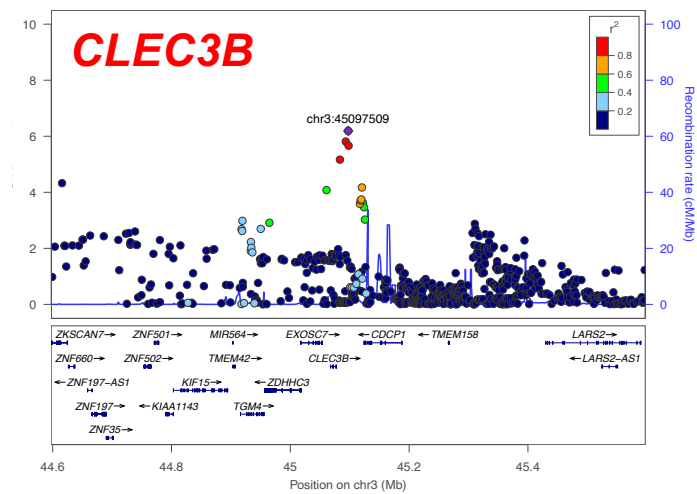

(b16)

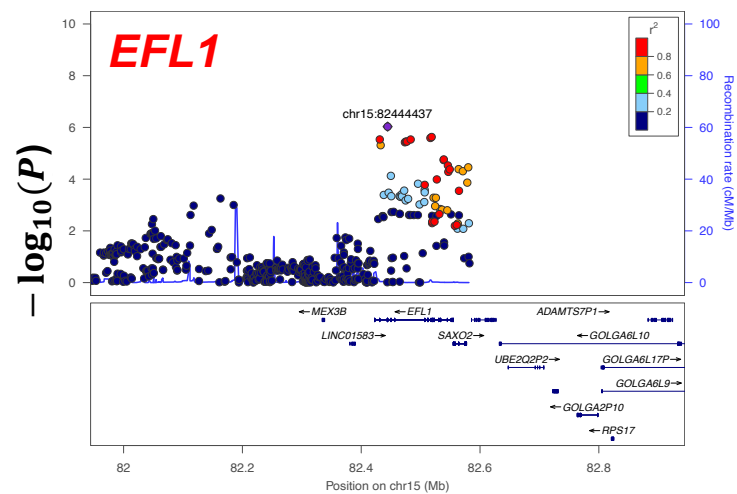

(b17)

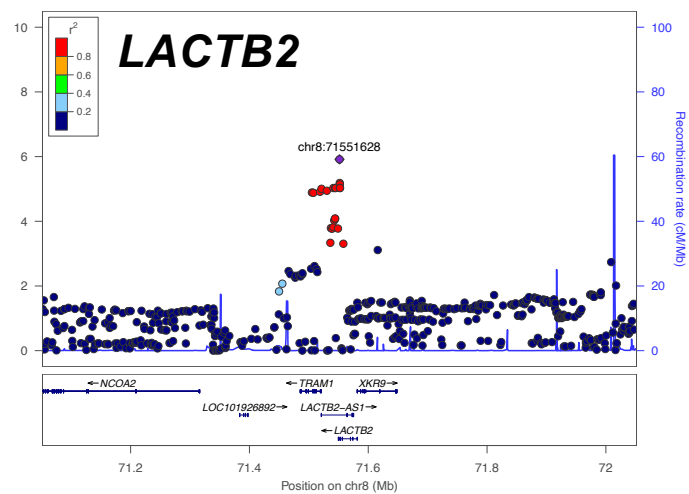

(b18)

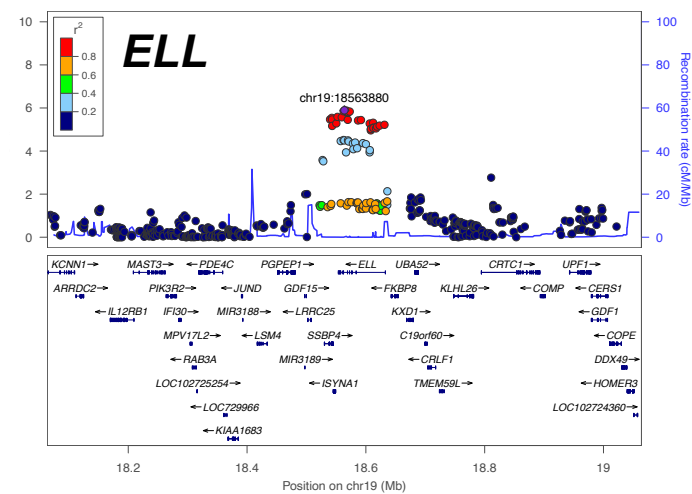

(b19)

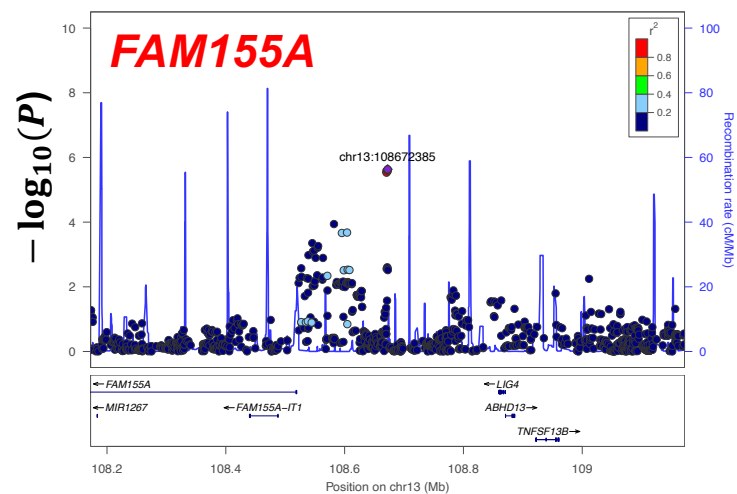

(b20)

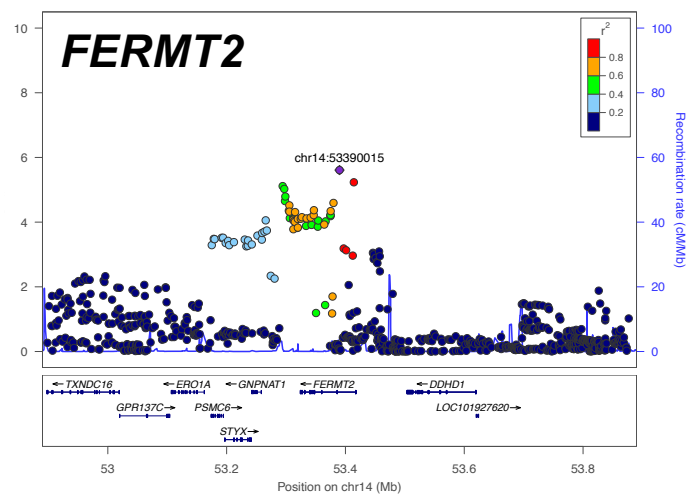

(b21)

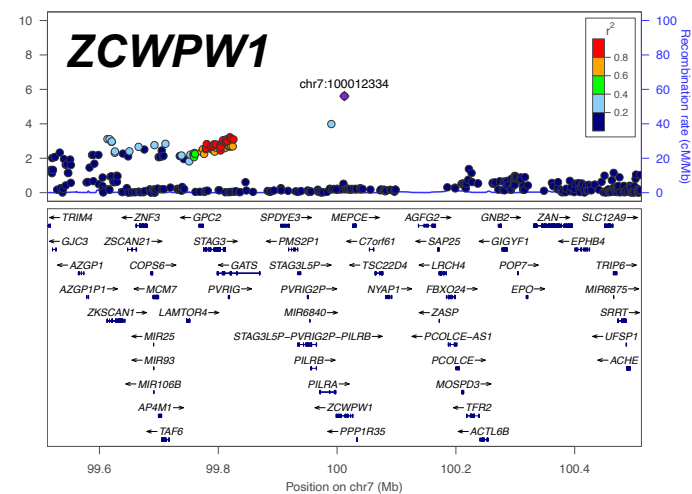

(b22)

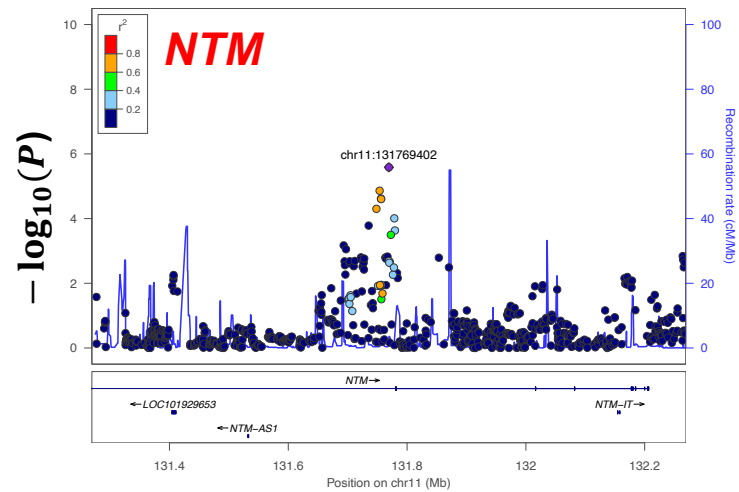

(b23)

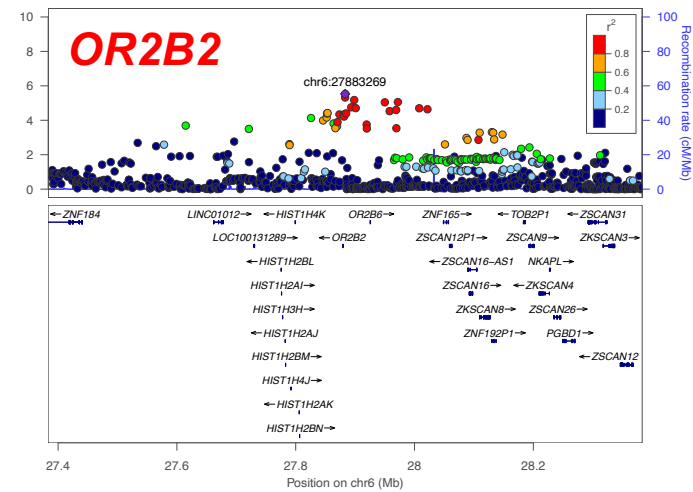

(b24)

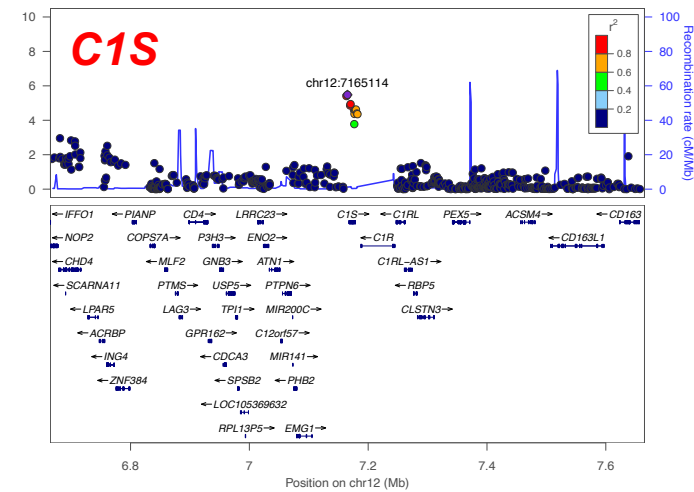

(b25)

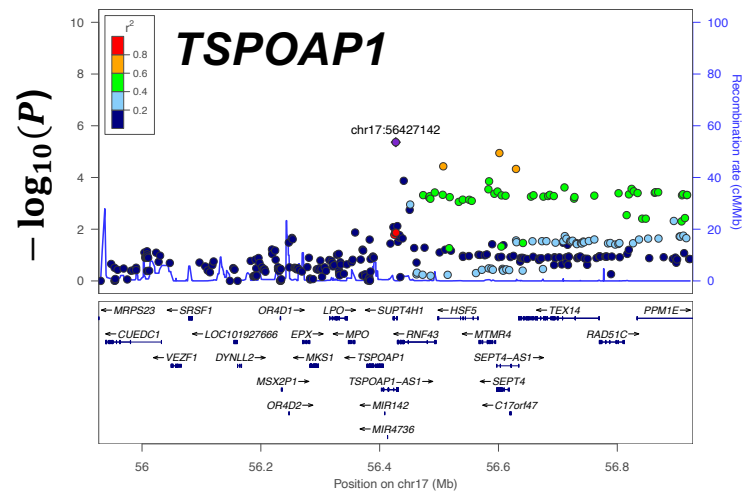

(b26)

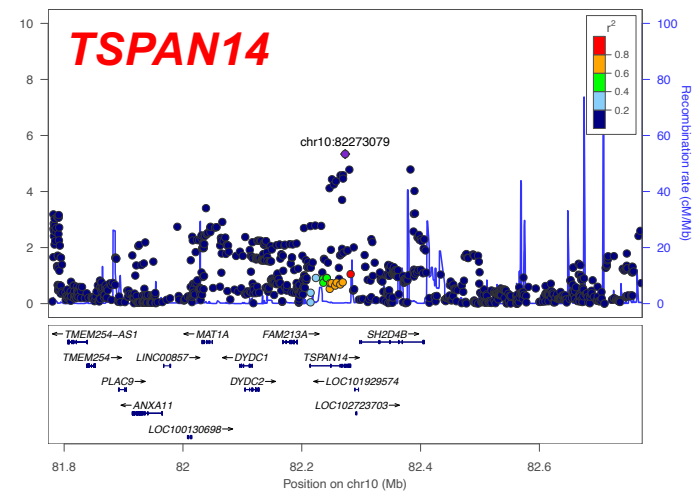

Supplement: Supplementary file 2 — Supplemental Figure 2 [file 41398_2021_1272_MOESM2_ESM.pdf]
