# Supplementary material for: Ethnic and trans-ethnic genome-wide association studies identify new loci influencing Japanese Alzheimer’s disease risk
Source: Transl Psychiatry. 2021 Mar 3;11:151. doi: 10.1038/s41398-021-01272-3 (PMC7925686; doi:10.1038/s41398-021-01272-3)
Supplement: Supplementary file 3 — Supplemental Table 1 [file 41398_2021_1272_MOESM3_ESM.pdf]

Table S1. Conditional logistic regression analysis on rs920608 for all SNPs with $P < 1.0 \times 10^{-4}$

SNP	Position (GRCh37)	Unconditional *P*	*P* conditional on rs920608
rs4282210	77,142,346	3.12×10^{-7}	0.81
rs1465923	77,136,211	8.15×10^{-7}	Colinear
rs10032423	77,140,733	9.64×10^{-7}	0.82
rs1866975	77,138,658	1.41×10^{-6}	0.84
rs7685696	77,139,510	1.51×10^{-6}	0.84
rs9991301	77,142,235	3.64×10^{-6}	0.79
rs76069026	77,133,175	6.60×10^{-6}	Colinear
Chr4:77166007	77,166,007	1.48×10^{-5}	0.83
rs61216514	77,163,722	1.56×10^{-5}	0.77
rs62300773	77,164,792	2.95×10^{-5}	0.92
rs72655564	77,169,876	4.05×10^{-5}	0.82

Abbreviations: Chr, chromosome; GRCh37, genome reference consortium human build 37; SNP, single-nucleotide polymorphism.
