# Supplementary material for: Ethnic and trans-ethnic genome-wide association studies identify new loci influencing Japanese Alzheimer’s disease risk
Source: Transl Psychiatry. 2021 Mar 3;11:151. doi: 10.1038/s41398-021-01272-3 (PMC7925686; doi:10.1038/s41398-021-01272-3)
Supplement: Supplementary file 4 — Supplemental Table 2 [file 41398_2021_1272_MOESM4_ESM.pdf]

**Table S2. The difference of MAF between EAS and other populations in rs920608 and rs1497526**

| SNP | Database | Population 1 | MAF of population 1 | Population 2 | MAF of population 2 | *P* -value (Fisher's exact test) |
|---|---|---|---|---|---|---|
| rs920608 | 1000Gp3 | EAS | 38/1008 = 0.038 | AFR | 144/1322 = 0.11 | 5.33E-11 |
| | | | | AMR | 11/694 = 0.016 | 7.72E-03 |
| | | | | EUR | 6/1006 = 0.0060 | 7.32E-07 |
| | | | | SAS | 1/978 = 0.021 | 3.48E-02 |
| | gnomAD v2.1.1 | | 67/1560 = 0.043 | AFR | 785/8706 = 0.090 | 5.93E-10 |
| | | | | AMR | 11/848 = 0.0130 | 5.21E-05 |
| | | | | EUR | 113/15422 = 0.0073 | <2.2E-16 |
| | | | | SAS | NA | NA |
| rs1497526 | 1000Gp3 | EAS | 82/1008 = 0.081 | AFR | 385/1322 = 0.29 | < 2.2E-16 |
| | | | | AMR | 63/694 = 0.091 | 0.54 |
| | | | | EUR | 46/1006 = 0.046 | 1.31E-03 |
| | | | | SAS | 74/978 = 0.076 | 0.68 |
| | gnomAD v2.1.1 | | 66/1550 = 0.043 | AFR | 2027/8466 = 0.24 | <2.2E-16 |
| | | | | AMR | 63/860 = 0.076 | 3.44E-03 |
| | | | | EUR | 495/15314 = 0.032 | 0.045 |
| | | | | SAS | NA | NA |
