# Supplementary material for: Ethnic and trans-ethnic genome-wide association studies identify new loci influencing Japanese Alzheimer’s disease risk
Source: Transl Psychiatry. 2021 Mar 3;11:151. doi: 10.1038/s41398-021-01272-3 (PMC7925686; doi:10.1038/s41398-021-01272-3)
Supplement: Supplementary file 5 — Supplemental Table 3 [file 41398_2021_1272_MOESM5_ESM.pdf]

Table S3. Twenty-six loci identified in a trans-ethnic meta-analysis

Locus	Chr	Position (GRCh37)	SNP	Meta-P (RE-HE)	gene	proxy SNP	GWAS-catalog	PMID	PMID (confirmed in Japanese)
1	19	45,407,788	rs7259620	2.35E−162	*APOE*	-	AD	30636644	458 articles
2	11	85,868,640	rs3851179	2.29E−19	*PICALM*	-	AD	19734902, 30979435, 25778476, 29777097	32991610, 23565137, 22935915
3	2	127,889,637	rs7561528	2.44E−19	*BIN1*	-	AD	25188341, 28714976, 29777097, 30617256	32991610, 23565137
4	8	27,466,157	rs1532276	8.7E−19	*CLU*	-	AD	19734903, 30805717, 19734902, 21627779, 21460841, 28714976, 25778476, 29777097, 24162737, 30617256, 31473137	32991610, 21447104
5	1	207,750,568	rs679515	3.02E−17	*CR1*	-	AD	25778476, 30617256, 29777097	32991610
6	11	60,021,948	rs1582763	2.56E−15	*MS4A4A*	-	AD	30617256, 29777097, 25778476, 21627779, 21460841	
7	11	121,456,061	rs117807585	1.73E−13	*SORL1*	-	AD	31473137, 30617256, 29777097, 25778476, 24162737, 23565137	23565137,19539718, 23455993, 31588051, 18685254
8	11	47,391,745	rs67472071	2.01E−9	*MADD*	-	AD	30805717	
9	6	32,386,619	rs4335021	2.72E−9	*HLA - DRA*	-	AD	30617256	
10	6	47,450,618	rs9473119	1.73E−7	*CD2AP*	-	AD	30617256, 29777097, 24162737, 21460841, 31473137	
11	16	70,701,411	rs7195572	1.83E−7	*MTSS1L*	-	Novel		
12	7	143,107,876	rs11762262	2.35E−7	*EPHA1*	-	AD	31473137, 30617256, 29777097, 25778476, 24162737, 21460841	
13	21	28,148,191	rs2830489	2.67E−7	*ADAMTS1*	-	AD	30820047	
14	14	92,933,893	rs11160069	2.91E−7	*SLC24A4*	-	AD	31473137, 30617256, 25778476, 24162737	
15	3	45,097,509	rs7618668	6.43E−7	*CLEC3B*	rs7626571	Novel		
16	15	82,444,437	rs905450	9.31E−7	*EFL1*	-	Novel		
17	8	71,551,628	rs13252043	1.22E−6	*LACTB2*	-	AD	29110684	
18	19	18,563,880	rs10405479	1.28E−6	*ELL*	-	AD	30820047	
19	13	108,672,385	rs9520713	2.33E−6	*FAM155A*	-	Novel		
20	14	53,390,015	rs74825460	2.46E−6	*FERMT2*	-	AD	27244899	
21	7	100,012,334	rs34919929	2.46E−6	*ZCWPW1*	-	AD	29777097, 30617256, 24162737	
22	11	131,769,402	rs9787911	2.59E−6	*NTM*	-	Novel		
23	6	27,883,269	rs1497525	2.96E−6	*OR2B2*	rs1497526	Novel		
24	12	7,165,114	rs7311672	3.32E−6	*C1S*	-	Novel		
25	17	56,427,142	rs2526376	4.33E−6	*TSPOAP1*	-	AD	30820047, 30617256, 28183528	
26	10	82,273,079	rs10748526	4.6E−6	*TSPAN14*	-	Novel		

Abbreviations: Chr, chromosome; GRCh37, genome reference consortium human build 37; SNP, single nucleotide polymorphism; RE-HE, Han and Eskin's modified random effects model; GWAS, genome-wide association study; PMID, PubMed ID
