# Supplementary material for: Ethnic and trans-ethnic genome-wide association studies identify new loci influencing Japanese Alzheimer’s disease risk
Source: Transl Psychiatry. 2021 Mar 3;11:151. doi: 10.1038/s41398-021-01272-3 (PMC7925686; doi:10.1038/s41398-021-01272-3)
Supplement: Supplementary file 6 — Supplemental Table 4 [file 41398_2021_1272_MOESM6_ESM.pdf]

Table S4. Quantitative trait locus (QTL) analysis for *FAM47E* and *OR2B2* SNPs

Gene	Rank	QT	Effect	SE	*P*
FAM47E	1	I-BIL[serum]	0.053	0.021	0.011
	2	MPV	0.117	0.048	0.014
	3	CRE[serum]	0.070	0.030	0.019
	4	D-BIL[serum]	0.088	0.040	0.027
	5	eGFR	-2.431	1.127	0.031
	6	GLU[serum]	8.330	4.092	0.042
	7	HbA1c	0.095	0.049	0.055
	8	ALP[serum]	14.252	8.188	0.082
	9	Mean red blood cell hemoglobin amount	0.172	0.103	0.095
	10	CHE[serum]	10.599	6.486	0.102
	11	Cystatin C	0.048	0.030	0.115
	12	TPHA[serum]	8.217	5.269	0.119
	13	GLU[plasma]	3.463	2.248	0.123
	14	FT4[serum]	-0.014	0.009	0.128
	15	Fe[serum]	-2.949	1.942	0.129
	16	IP[serum]	0.040	0.028	0.157
	17	Mean red blood cell volume	0.342	0.265	0.196
	18	Urine specific gravity	0.000	0.000	0.198
	19	AMY[serum]	5.630	4.549	0.216
	20	Leukocyte count	1.336	1.084	0.218
	21	Hemoglobin amount	0.099	0.085	0.246
	22	Platelet count	-0.368	0.336	0.272
	23	Basophil%	0.019	0.018	0.279
	24	nonHDL-C	-2.370	2.233	0.289
	25	LDL cholesterol[serum]	-1.838	1.806	0.309
	26	Hematocrit	0.240	0.237	0.310
	27	A/G ratio	-0.015	0.016	0.358
	28	FT3[serum]	-0.018	0.021	0.389
	29	TG[serum]	3.497	4.090	0.393
	30	Monocyte%	0.103	0.128	0.423
	31	Na[serum] Na[serum]	-0.137	0.172	0.426
	32	Vitamin B1	-3.619	4.584	0.430
	33	BNP	6.055	7.915	0.444
	34	K[serum]	0.017	0.023	0.462
	35	Cl[serum]	-0.143	0.197	0.468
	36	T-CHO[serum]	-1.447	2.132	0.497
	37	ALT[serum]	-0.816	1.254	0.515
	38	AST	-1.108	1.937	0.567
	39	CK[serum]	3.884	7.526	0.606
	40	Vitamin B12	31.659	65.805	0.630
	41	Urine PH	0.015	0.037	0.694
	42	Ca[serum]	0.010	0.028	0.711
	43	r-GT	0.874	2.399	0.716
	44	HDL cholesterol[serum]	-0.295	0.886	0.739
	45	Fibrinogen	2.033	6.115	0.740
	46	Red blood cell count	0.923	2.797	0.741
	47	Mean red blood cell hemoglobin concentration	0.016	0.055	0.768
	48	Folic acid	-0.622	2.163	0.774

	49	Neutrophil%	-0.121	0.555	0.828
	50	ALB[serum]	-0.004	0.029	0.887
	51	Total Homocysteine	-0.059	0.490	0.904
	52	TP[serum]	0.004	0.033	0.910
	53	UA[serum]	0.013	0.120	0.911
	54	UN[serum]	-0.047	0.446	0.917
	55	Eosinophil%	0.013	0.126	0.920
	56	TSH[serum]	0.004	0.094	0.962
	57	T-BIL[serum]	0.001	0.021	0.970
	58	Lymphocytes%	-0.015	0.496	0.977
	59	CRP[serum]	0.003	0.110	0.980
	60	LDH[serum]	0.006	4.194	0.999
OR2B2	1	TP[serum]	-0.045	0.018	0.012
	2	MPV	0.059	0.026	0.023
	3	UN[serum]	-0.457	0.242	0.059
	4	ALB[serum]	-0.024	0.016	0.122
	5	LDH[serum]	-3.274	2.282	0.151
	6	UA[serum]	-0.090	0.065	0.163
	7	D-BIL[serum]	0.027	0.022	0.208
	8	CRE[serum]	0.020	0.016	0.215
	9	HDL cholesterol[serum]	-0.576	0.478	0.229
	10	Ca[serum]	-0.018	0.015	0.244
	11	TG[serum]	2.551	2.220	0.251
	12	Basophil%	0.011	0.010	0.265
	13	FT4[serum]	0.005	0.005	0.279
	14	Hematocrit	-0.134	0.128	0.297
	15	Vitamin B12	36.569	35.177	0.299
	16	Hemoglobin amount	-0.045	0.046	0.332
	17	TSH[serum]	-0.049	0.051	0.333
	18	ALT[serum]	-0.653	0.680	0.337
	19	HbA1c	-0.025	0.027	0.341
	20	Vitamin B1	-2.190	2.452	0.372
	21	Total homocysteine	0.223	0.254	0.380
	22	Red blood cell count	-1.315	1.514	0.385
	23	nonHDL-C	1.066	1.237	0.389
	24	K[serum]	-0.011	0.013	0.402
	25	CHE[serum]	-2.862	3.435	0.405
	26	eGFR	0.500	0.612	0.413
	27	T-CHO[serum]	-0.933	1.158	0.421
	28	Lymphocytes%	-0.215	0.268	0.422
	29	T-BIL[serum]	0.008	0.011	0.456
	30	Folic acid	-0.847	1.150	0.462
	31	Eosinophil%	0.049	0.068	0.470
	32	IP[serum]	-0.010	0.015	0.489
	33	Urine specific gravity	0.000	0.000	0.509
	34	AST	-0.666	1.053	0.527
	35	ALP[serum]	-2.795	4.442	0.529
	36	Cl[serum]	0.067	0.107	0.530
	37	GLU[serum]	-1.371	2.208	0.535
	38	Neutrophil%	0.172	0.300	0.568

39	Urine PH	0.011	0.020	0.585
40	GLU[plasma]	-0.645	1.214	0.595
41	CK[serum]	2.058	4.080	0.614
42	r-GT	0.446	1.288	0.729
43	LDL cholesterol[serum]	-0.311	0.972	0.749
44	Leukocyte count	-0.184	0.585	0.753
45	Na[serum] Na[serum]	0.028	0.094	0.764
46	TPHA[serum]	-0.831	2.784	0.765
47	I-BIL[serum]	-0.003	0.011	0.789
48	BNP	1.072	4.349	0.805
49	Cystatin C	-0.003	0.016	0.833
50	Fibrinogen	0.484	3.130	0.877
51	A/G ratio	-0.001	0.009	0.887
52	Mean red blood cell hemoglobin concentration	-0.004	0.030	0.894
53	Mean red blood cell hemoglobin amount	-0.007	0.056	0.902
54	Mean red blood cell volume	-0.014	0.144	0.923
55	AMY[serum]	0.220	2.366	0.926
56	Platelet count	0.013	0.182	0.943
57	Fe[serum]	-0.060	1.002	0.953
58	FT3[serum]	0.001	0.011	0.955
59	Monocyte%	0.003	0.069	0.964
60	CRP[serum]	0.002	0.059	0.969
