# Supplementary material for: Ethnic and trans-ethnic genome-wide association studies identify new loci influencing Japanese Alzheimer’s disease risk
Source: Transl Psychiatry. 2021 Mar 3;11:151. doi: 10.1038/s41398-021-01272-3 (PMC7925686; doi:10.1038/s41398-021-01272-3)
Supplement: Supplementary file 7 — Supplemental Table 5 [file 41398_2021_1272_MOESM7_ESM.pdf]

Table S5. Association analysis for *FAM47E* SNPs and *OR2B2* SNPs in the IGAP data

gene	chr	pos	rs#	EA	NEA	beta	SE	*P*
FAM47E	4	77221027	chr4:77221027:I	A	AT	0.0434	0.0146	0.002928
FAM47E	4	77221028	chr4:77221028:I	A	ATT	0.0433	0.0146	0.002972
FAM47E	4	77221028	rs62300830	A	T	0.0424	0.0145	0.003562
FAM47E	4	77219057	rs6841455	T	G	0.0453	0.0161	0.004891
FAM47E	4	77212286	rs115784532	A	G	-0.1492	0.0541	0.005817
FAM47E	4	77219402	rs6830162	T	G	-0.0436	0.016	0.006299
FAM47E	4	77221247	rs72655601	T	C	0.0433	0.0168	0.009896
FAM47E	4	77220837	rs12506745	T	G	0.0369	0.0144	0.01047
FAM47E	4	77227676	rs56049812	A	C	0.0341	0.0145	0.01851
FAM47E	4	77174933	rs187242602	C	G	0.1729	0.0802	0.03121
FAM47E	4	77212217	rs11935444	T	C	0.0304	0.0144	0.03545
FAM47E	4	77217924	rs7655812	A	T	-0.0349	0.0166	0.03556
FAM47E	4	77211865	rs77241262	A	G	0.0378	0.0182	0.03739
FAM47E	4	77143264	rs114096978	T	C	-0.1002	0.0482	0.03766
FAM47E	4	77208722	chr4:77208722:I	T	TA	0.0351	0.0169	0.03782
FAM47E	4	77211819	rs138861398	A	C	0.0354	0.0171	0.03831
FAM47E	4	77208973	rs2197103	A	T	0.0347	0.0169	0.03966
FAM47E	4	77208601	rs6532329	T	C	0.0346	0.0169	0.0403
FAM47E	4	77208412	rs6532327	T	C	-0.0346	0.0169	0.04058
FAM47E	4	77208482	rs6532328	T	C	0.0346	0.0169	0.04059
FAM47E	4	77211690	rs11729096	A	G	0.0346	0.0169	0.0407
FAM47E	4	77217812	rs1876533	T	C	0.0293	0.0144	0.041
FAM47E	4	77210143	chr4:77210143:D	A	ACAGATC	0.035	0.0172	0.0415
FAM47E	4	77209750	rs115292116	T	C	0.0344	0.0169	0.04205
FAM47E	4	77207955	chr4:77207955:D	CT	C	-0.0343	0.0169	0.04206
FAM47E	4	77210255	rs62300827	A	G	-0.0338	0.017	0.04661
FAM47E	4	77156782	rs116361144	C	G	-0.1763	0.0902	0.05066
FAM47E	4	77210214	rs143011124	A	C	-0.0332	0.0172	0.05303
FAM47E	4	77223374	rs79265085	A	G	0.1571	0.0837	0.06063
FAM47E	4	77208191	rs4859660	T	G	-0.0316	0.0168	0.06078
FAM47E	4	77148509	rs1583942	A	C	0.5954	0.3179	0.06108
FAM47E	4	77161566	rs79144120	A	G	0.6038	0.3281	0.06571
FAM47E	4	77212517	rs75191812	T	C	-0.0955	0.0523	0.06776
FAM47E	4	77165489	rs192185572	A	G	0.191	0.1058	0.07105
FAM47E	4	77213829	rs972113	A	T	0.0257	0.0144	0.07354
FAM47E	4	77140007	rs184225087	A	G	-0.46	0.258	0.0746
FAM47E	4	77227485	rs3733249	A	G	0.0369	0.0207	0.07463
FAM47E	4	77163348	rs112317196	T	C	-0.5889	0.3312	0.07538
FAM47E	4	77166624	rs7670989	A	T	0.5878	0.3311	0.07582
FAM47E	4	77208107	rs4859442	A	G	-0.0253	0.0144	0.07886
FAM47E	4	77205745	rs2869881	T	C	0.0309	0.0177	0.0803
FAM47E	4	77210387	rs59738370	A	T	0.0252	0.0144	0.08121
FAM47E	4	77208436	rs7698049	A	G	-0.0251	0.0144	0.08146
FAM47E	4	77205338	rs7662031	T	C	0.0303	0.0177	0.08707
FAM47E	4	77205746	rs2869882	A	T	-0.03	0.0177	0.08928
FAM47E	4	77205319	rs62300825	A	G	0.0298	0.0177	0.09173
FAM47E	4	77215852	rs6852266	T	C	0.4176	0.2536	0.09966
FAM47E	4	77204398	chr4:77204398:D	CAT	C	-0.0291	0.0176	0.09974
FAM47E	4	77190768	chr4:77190768:D	T	TA	-0.039	0.0239	0.1028
FAM47E	4	77206895	rs147055103	T	C	0.0272	0.0168	0.1057
FAM47E	4	77178236	rs117724978	A	G	0.2889	0.1849	0.1182
FAM47E	4	77148153	chr4:77148153:I	T	TTTTG	0.0996	0.0649	0.1247
FAM47E	4	77222326	rs187268711	A	C	-0.2213	0.1447	0.1262

FAM47E	4	77174597	rs76220525	T	C	0.5097	0.3368	0.1302
FAM47E	4	77174991	rs111921856	T	G	0.5075	0.3367	0.1317
FAM47E	4	77176095	rs17001718	A	G	0.5043	0.3363	0.1338
FAM47E	4	77176766	rs147963209	A	G	0.5042	0.3363	0.1339
FAM47E	4	77176786	rs80276579	T	G	-0.5042	0.3363	0.1339
FAM47E	4	77218368	rs191040296	A	T	-0.3601	0.2463	0.1438
FAM47E	4	77190011	rs140609514	T	C	0.22	0.1522	0.1482
FAM47E	4	77212925	rs17001751	A	G	-0.4952	0.3426	0.1483
FAM47E	4	77184555	rs114372776	T	C	-0.4939	0.343	0.1499
FAM47E	4	77198788	rs17001742	A	G	-0.4944	0.3439	0.1505
FAM47E	4	77195073	rs7693209	T	C	-0.494	0.3437	0.1506
FAM47E	4	77223812	rs149999828	C	G	-0.1776	0.1242	0.1529
FAM47E	4	77194221	rs768980	A	G	-0.4915	0.3448	0.154
FAM47E	4	77198363	rs6810903	T	C	0.0251	0.0177	0.155
FAM47E	4	77212363	rs6813244	A	C	-0.4864	0.3422	0.1552
FAM47E	4	77172716	rs75886691	T	C	0.0537	0.0378	0.1557
FAM47E	4	77197438	chr4:77197438:D	G	GA	0.4903	0.3466	0.1572
FAM47E	4	77202019	rs17237174	T	C	-0.0249	0.0176	0.1572
FAM47E	4	77181983	rs78146498	T	C	-0.482	0.3422	0.1589
FAM47E	4	77196476	rs17236661	T	G	-0.0245	0.0177	0.165
FAM47E	4	77196220	chr4:77196220:I	A	AT	-0.0245	0.0177	0.1653
FAM47E	4	77196586	rs75019383	A	G	-0.0828	0.0599	0.1665
FAM47E	4	77195106	rs28864163	C	G	0.0244	0.0177	0.1675
FAM47E	4	77193208	rs77937770	A	T	-0.024	0.0177	0.174
FAM47E	4	77137095	rs150563545	A	G	-0.1274	0.0938	0.1744
FAM47E	4	77197845	rs28698332	A	G	-0.0236	0.0175	0.176
FAM47E	4	77196188	rs6822055	T	C	-0.0231	0.0175	0.1855
FAM47E	4	77178121	rs144847024	T	G	0.1422	0.1087	0.1909
FAM47E	4	77202861	rs1530297	A	G	0.0187	0.0144	0.1942
FAM47E	4	77176018	rs183900321	A	G	-0.3743	0.2894	0.1959
FAM47E	4	77191952	rs28618267	A	G	0.0224	0.0175	0.1999
FAM47E	4	77203413	rs1969148	C	G	0.0184	0.0144	0.2
FAM47E	4	77190649	rs28506049	A	G	-0.022	0.0177	0.2127
FAM47E	4	77218240	rs1508335	A	G	-0.0181	0.0146	0.2153
FAM47E	4	77174072	rs13149410	T	C	-0.0504	0.0408	0.2163
FAM47E	4	77144016	rs57228398	A	G	0.4766	0.3866	0.2176
FAM47E	4	77175166	rs146201191	T	C	0.2133	0.173	0.2176
FAM47E	4	77175335	rs142588499	T	G	0.2109	0.173	0.2229
FAM47E	4	77225659	rs12501628	T	C	-0.0184	0.0153	0.2275
FAM47E	4	77186642	rs17236179	A	G	0.0203	0.0175	0.2452
FAM47E	4	77203043	rs1969149	T	C	-0.0165	0.0144	0.2507
FAM47E	4	77183300	rs7695720	A	C	-0.02	0.0175	0.2522
FAM47E	4	77183434	rs7654472	T	C	-0.0199	0.0175	0.2541
FAM47E	4	77202812	rs1530296	A	G	0.0163	0.0144	0.2553
FAM47E	4	77202882	rs1530298	A	C	-0.0163	0.0144	0.2579
FAM47E	4	77185879	rs28649105	A	G	-0.0198	0.0175	0.2583
FAM47E	4	77178682	rs114978691	T	C	-1.1429	1.0192	0.2621
FAM47E	4	77204500	rs1542096	T	C	-0.0167	0.015	0.2649
FAM47E	4	77188418	rs17001729	C	G	-0.0195	0.0175	0.2663
FAM47E	4	77188327	rs28604357	T	G	-0.0194	0.0175	0.2666
FAM47E	4	77220381	rs141846617	T	C	0.1993	0.1794	0.2666
FAM47E	4	77188288	rs28597785	C	G	0.0194	0.0175	0.2669
FAM47E	4	77177047	rs17001727	A	G	-0.3234	0.2922	0.2684
FAM47E	4	77187916	rs7664593	T	C	0.0193	0.0175	0.2689
FAM47E	4	77188766	rs28498588	A	T	-0.0193	0.0175	0.2697

FAM47E	4	77189380	rs10025494	A	G	-0.0192	0.0175	0.2736
FAM47E	4	77188058	rs10019829	A	G	-0.0191	0.0175	0.275
FAM47E	4	77195732	rs4859440	T	C	0.0156	0.0143	0.2751
FAM47E	4	77217605	rs186469969	A	G	-0.1597	0.1472	0.2779
FAM47E	4	77189418	rs62300803	T	C	0.019	0.0175	0.2788
FAM47E	4	77203859	chr4:77203859:D	CT	C	0.0152	0.0144	0.2899
FAM47E	4	77199209	rs183961659	A	T	0.2036	0.1925	0.2902
FAM47E	4	77185176	rs72860111	A	T	0.0282	0.0271	0.2988
FAM47E	4	77185194	rs72860114	T	C	-0.0282	0.0271	0.2989
FAM47E	4	77176552	rs7671407	A	G	0.016	0.0157	0.3083
FAM47E	4	77198566	rs6858344	A	G	0.0152	0.0149	0.3101
FAM47E	4	77144259	rs1114091	A	G	-0.014	0.0144	0.3306
FAM47E	4	77182033	rs2904221	T	C	-0.0152	0.0156	0.3309
FAM47E	4	77210937	rs1837872	T	G	0.3611	0.3725	0.3323
FAM47E	4	77189667	rs182113748	A	G	-0.1594	0.1662	0.3374
FAM47E	4	77222933	rs10019831	A	C	0.0219	0.0231	0.341
FAM47E	4	77197651	rs28507491	A	G	0.0144	0.0152	0.342
FAM47E	4	77143714	rs142931819	T	C	0.0755	0.0796	0.3427
FAM47E	4	77220470	rs145515199	T	C	-0.0622	0.0658	0.3442
FAM47E	4	77182830	rs60319873	A	G	-0.9478	1.0088	0.3475
FAM47E	4	77136408	rs182932808	T	C	0.065	0.0694	0.349
FAM47E	4	77149785	rs7675593	T	C	-0.0131	0.0142	0.3548
FAM47E	4	77150642	rs62303008	T	C	-0.013	0.0142	0.3601
FAM47E	4	77194803	rs12503821	A	G	0.023	0.0251	0.3606
FAM47E	4	77152680	chr4:77152680:D	A	AAAAC	-0.0135	0.0149	0.3636
FAM47E	4	77196143	rs12646723	T	C	0.0228	0.0251	0.3654
FAM47E	4	77191596	rs4859654	A	G	0.0128	0.0144	0.3734
FAM47E	4	77222932	rs10019830	A	T	0.0203	0.0229	0.3739
FAM47E	4	77149293	rs1911738	T	C	-0.0126	0.0142	0.3743
FAM47E	4	77146157	rs4306982	A	C	-0.0139	0.0157	0.3751
FAM47E	4	77137580	rs62303000	A	T	0.0128	0.0144	0.3752
FAM47E	4	77145983	rs7665857	A	G	-0.0139	0.0157	0.3773
FAM47E	4	77136211	rs1465923	T	C	0.0655	0.0745	0.3793
FAM47E	4	77198791	rs11934863	A	G	-0.0126	0.0144	0.3814
FAM47E	4	77146052	rs7667447	T	C	-0.0138	0.0157	0.3815
FAM47E	4	77152647	rs4859433	T	C	-0.0122	0.0142	0.389
FAM47E	4	77180934	rs114184822	A	G	-0.0605	0.0704	0.3905
FAM47E	4	77197741	chr4:77197741:D	CCCAA	C	0.0124	0.0144	0.3905
FAM47E	4	77170528	rs116704525	A	G	-0.1914	0.223	0.3907
FAM47E	4	77140297	rs59473442	A	T	-0.0123	0.0144	0.3933
FAM47E	4	77145954	rs7667229	A	C	0.0122	0.0143	0.394
FAM47E	4	77153137	rs4859640	T	C	-0.0121	0.0142	0.3941
FAM47E	4	77153836	rs143052491	T	C	0.1132	0.1334	0.3963
FAM47E	4	77200799	rs11946804	T	C	-0.0122	0.0144	0.3966
FAM47E	4	77200875	rs11933305	A	G	0.0122	0.0144	0.3966
FAM47E	4	77199941	rs4859441	T	C	-0.0122	0.0144	0.3967
FAM47E	4	77200805	rs11946806	T	C	-0.0122	0.0144	0.3967
FAM47E	4	77141118	rs56153157	A	G	0.0122	0.0144	0.3973
FAM47E	4	77199211	rs3817118	A	C	-0.0213	0.0252	0.3983
FAM47E	4	77203855	chr4:77203855:D	G	GC	-0.0123	0.0146	0.3988
FAM47E	4	77136827	rs55889442	C	G	-0.0122	0.0145	0.4003
FAM47E	4	77142050	rs7675963	C	G	-0.0122	0.0145	0.4013
FAM47E	4	77138460	rs920608	A	C	0.0624	0.0745	0.4021
FAM47E	4	77176281	rs7666265	A	G	0.0151	0.0181	0.4039
FAM47E	4	77145713	rs6848690	A	T	0.0143	0.0171	0.4043

FAM47E	4	77194881	rs4859657	T	C	-0.0119	0.0144	0.4069
FAM47E	4	77199496	rs191258934	T	C	-0.4197	0.5112	0.4116
FAM47E	4	77197882	rs13123108	C	G	-0.0118	0.0144	0.4134
FAM47E	4	77197397	rs28636815	A	G	-0.0127	0.0155	0.4153
FAM47E	4	77155742	rs72858533	C	G	0.0197	0.0243	0.4163
FAM47E	4	77190932	rs1876541	T	C	0.0117	0.0144	0.4167
FAM47E	4	77199414	rs964051	A	C	-0.0117	0.0144	0.4169
FAM47E	4	77198571	rs7699714	A	G	-0.0116	0.0144	0.4217
FAM47E	4	77142346	rs4282210	A	C	0.0593	0.0741	0.4234
FAM47E	4	77155728	rs73828720	C	G	0.0193	0.0243	0.4252
FAM47E	4	77169876	rs72655564	A	C	0.0192	0.0241	0.4268
FAM47E	4	77196500	rs1441914	T	C	-0.0114	0.0144	0.4276
FAM47E	4	77165893	rs4859645	A	C	-0.0143	0.018	0.4286
FAM47E	4	77197396	rs28689064	T	C	0.0123	0.0155	0.4299
FAM47E	4	77172595	rs1441908	T	C	-0.014	0.018	0.4361
FAM47E	4	77164204	rs3910643	T	C	-0.0875	0.1128	0.4376
FAM47E	4	77214858	rs114816233	T	C	0.0389	0.0502	0.4381
FAM47E	4	77145707	chr4:77145707:I	CT	C	0.0261	0.0339	0.4416
FAM47E	4	77190902	rs1876540	T	C	0.0111	0.0144	0.4421
FAM47E	4	77166493	rs28459239	A	G	-0.0361	0.047	0.4423
FAM47E	4	77192838	rs3733251	A	C	0.0148	0.0193	0.4432
FAM47E	4	77136858	rs78737354	T	C	0.0193	0.0252	0.444
FAM47E	4	77187100	chr4:77187100:I	T	TA	-0.0193	0.0252	0.445
FAM47E	4	77150086	chr4:77150086:I	CA	C	0.1057	0.14	0.4503
FAM47E	4	77197520	chr4:77197520:D	T	TG	0.0115	0.0153	0.451
FAM47E	4	77197674	rs28578510	T	C	0.0114	0.0151	0.451
FAM47E	4	77197517	chr4:77197517:I	G	GT	-0.0115	0.0153	0.4517
FAM47E	4	77196848	rs6823078	A	G	0.0107	0.0144	0.4553
FAM47E	4	77194997	rs4859439	T	G	-0.0107	0.0144	0.4571
FAM47E	4	77195318	rs1583944	T	C	-0.0107	0.0144	0.4576
FAM47E	4	77193449	rs1441910	T	C	0.0106	0.0144	0.461
FAM47E	4	77193742	rs1441913	A	C	-0.0106	0.0144	0.461
FAM47E	4	77194684	rs4859656	T	C	-0.0106	0.0144	0.4611
FAM47E	4	77194670	rs4859438	T	G	-0.0106	0.0144	0.4613
FAM47E	4	77194664	rs4859655	T	G	0.0106	0.0144	0.4616
FAM47E	4	77190221	rs736636	A	T	-0.0106	0.0144	0.4622
FAM47E	4	77214031	rs115543498	A	C	-0.0984	0.1339	0.4622
FAM47E	4	77190680	rs2869870	A	G	-0.0106	0.0144	0.4629
FAM47E	4	77172511	rs1441907	T	C	-0.0133	0.0181	0.4637
FAM47E	4	77194246	rs768979	A	G	-0.0105	0.0144	0.4638
FAM47E	4	77194092	rs12643334	T	G	-0.0105	0.0144	0.4656
FAM47E	4	77196034	rs7665023	T	C	0.0104	0.0144	0.4673
FAM47E	4	77195110	rs72655587	T	C	0.014	0.0193	0.4674
FAM47E	4	77192688	rs3733253	T	G	-0.0104	0.0144	0.4685
FAM47E	4	77155703	rs73828719	C	G	0.014	0.0194	0.4691
FAM47E	4	77193136	rs1441909	A	G	0.0104	0.0144	0.4694
FAM47E	4	77192868	rs3733250	A	G	-0.0104	0.0144	0.4696
FAM47E	4	77192388	rs3821980	T	G	0.0104	0.0144	0.4706
FAM47E	4	77191422	rs59864739	A	G	-0.0103	0.0144	0.4721
FAM47E	4	77188339	rs72860117	A	T	0.0139	0.0193	0.4726
FAM47E	4	77191316	rs61595008	T	C	0.0103	0.0144	0.473
FAM47E	4	77193649	rs66820534	A	T	-0.0138	0.0193	0.473
FAM47E	4	77191260	rs60876302	A	T	0.0103	0.0144	0.4731
FAM47E	4	77192063	rs13151765	T	G	0.0103	0.0144	0.4735
FAM47E	4	77191049	rs1876543	T	C	0.0103	0.0144	0.4742

FAM47E	4	77191921	rs13119644	A	C	-0.0103	0.0144	0.4746
FAM47E	4	77190733	rs2869871	T	C	0.0103	0.0144	0.475
FAM47E	4	77191003	rs1876542	T	G	-0.0103	0.0144	0.4756
FAM47E	4	77190269	rs736634	T	C	0.0102	0.0144	0.4793
FAM47E	4	77166007	chr4:77166007:D	CAT	C	0.017	0.0242	0.4813
FAM47E	4	77201080	rs67373731	A	T	-0.0136	0.0193	0.4814
FAM47E	4	77163722	rs61216514	A	G	-0.017	0.0242	0.4815
FAM47E	4	77190069	rs1876539	T	C	-0.0101	0.0144	0.4831
FAM47E	4	77189984	rs1876538	T	C	0.01	0.0144	0.485
FAM47E	4	77175966	rs713097	A	G	0.0099	0.0142	0.4881
FAM47E	4	77208296	rs182058414	T	C	0.1398	0.2023	0.4896
FAM47E	4	77169394	rs6817956	T	C	0.0102	0.0148	0.491
FAM47E	4	77197223	rs1837870	A	C	-0.0104	0.0151	0.4915
FAM47E	4	77219229	chr4:77219229:D	A	ACACT	-0.0392	0.0571	0.492
FAM47E	4	77211104	rs1837871	A	G	0.0137	0.0199	0.4923
FAM47E	4	77158193	rs145143663	T	C	0.0215	0.0316	0.4968
FAM47E	4	77198465	rs6858114	A	G	0.01	0.0148	0.4977
FAM47E	4	77191355	chr4:77191355:D	T	TC	-0.0097	0.0144	0.4997
FAM47E	4	77173299	rs4859647	T	C	-0.0118	0.018	0.5116
FAM47E	4	77202310	rs115777572	C	G	0.0266	0.0411	0.5183
FAM47E	4	77181544	rs34034456	T	G	-0.0094	0.0147	0.5204
FAM47E	4	77164468	rs4859642	A	C	-0.0095	0.0148	0.5214
FAM47E	4	77164549	rs4859643	C	G	-0.0095	0.0148	0.5216
FAM47E	4	77212152	rs6829982	A	G	0.2383	0.3745	0.5245
FAM47E	4	77188132	rs4859650	A	G	0.0091	0.0144	0.5249
FAM47E	4	77186596	rs17236158	C	G	0.0092	0.0145	0.5267
FAM47E	4	77227177	rs79266183	T	C	0.105	0.1673	0.5301
FAM47E	4	77183931	rs79393144	T	C	-0.0158	0.0252	0.5314
FAM47E	4	77183646	rs74889910	A	G	-0.0157	0.0252	0.5319
FAM47E	4	77176524	rs7655112	T	C	-0.0091	0.0147	0.5349
FAM47E	4	77171957	rs7657929	T	G	-0.011	0.0181	0.5423
FAM47E	4	77139510	rs7685696	A	G	0.0087	0.0144	0.5453
FAM47E	4	77198637	rs6811535	T	C	0.0091	0.0151	0.5458
FAM47E	4	77171728	rs1372408	A	G	0.0109	0.0181	0.5466
FAM47E	4	77142235	rs9991301	T	C	-0.0087	0.0145	0.5491
FAM47E	4	77185529	rs1876536	C	G	-0.0086	0.0144	0.55
FAM47E	4	77140733	rs10032423	T	C	-0.0086	0.0144	0.5522
FAM47E	4	77138658	rs1866975	T	C	-0.0086	0.0144	0.5525
FAM47E	4	77155193	rs6814385	A	C	-0.0126	0.0213	0.5545
FAM47E	4	77156159	rs151157238	T	C	-0.1297	0.2209	0.5572
FAM47E	4	77188240	rs4859651	A	G	0.0084	0.0144	0.5605
FAM47E	4	77199913	rs28628748	A	G	0.0085	0.0146	0.5614
FAM47E	4	77198986	rs6812193	T	C	0.0085	0.0146	0.5616
FAM47E	4	77175687	rs55870158	A	G	-0.0082	0.0142	0.5633
FAM47E	4	77214327	rs74914162	A	G	-0.2521	0.4383	0.5652
FAM47E	4	77198054	rs6854006	T	C	0.0084	0.0146	0.5655
FAM47E	4	77187556	rs11097314	A	G	-0.0082	0.0144	0.5701
FAM47E	4	77162824	rs2904216	A	G	0.0102	0.0181	0.5739
FAM47E	4	77137917	rs111467309	C	G	0.0444	0.079	0.5742
FAM47E	4	77205471	rs189194623	T	G	-0.0716	0.1277	0.5752
FAM47E	4	77185997	rs1876537	T	C	-0.0081	0.0144	0.5753
FAM47E	4	77177817	rs2289514	A	G	-0.0081	0.0146	0.5775
FAM47E	4	77155507	rs6837231	T	C	-0.0099	0.0179	0.5818
FAM47E	4	77227354	rs79734427	T	C	0.0686	0.1252	0.5838
FAM47E	4	77186672	rs60397621	T	C	0.0078	0.0144	0.5856

FAM47E	4	77186415	rs17308336	A	C	0.0078	0.0144	0.5859
FAM47E	4	77175112	rs28718095	A	G	0.0114	0.0212	0.5915
FAM47E	4	77170728	chr4:77170728:I	A	AT	0.0301	0.0562	0.5929
FAM47E	4	77179518	rs6849069	T	G	0.0078	0.0146	0.5947
FAM47E	4	77159996	rs1441917	A	G	0.0096	0.018	0.5957
FAM47E	4	77222110	rs79854036	T	C	-0.0691	0.1302	0.5958
FAM47E	4	77175743	rs717239	A	G	-0.0075	0.0142	0.5973
FAM47E	4	77196677	rs1946959	A	G	0.0077	0.0146	0.5987
FAM47E	4	77155588	rs6854218	T	G	0.0111	0.0212	0.602
FAM47E	4	77159925	rs1441918	T	C	0.0093	0.018	0.607
FAM47E	4	77180816	rs12646634	T	G	-0.0129	0.0252	0.6075
FAM47E	4	77217955	rs17001764	A	G	-0.227	0.4419	0.6075
FAM47E	4	77162658	rs2904215	C	G	0.0092	0.018	0.6108
FAM47E	4	77182004	rs2869865	T	G	0.0074	0.0146	0.6111
FAM47E	4	77163130	rs12501795	A	G	0.0092	0.018	0.6112
FAM47E	4	77158348	rs35177915	A	G	-0.0091	0.018	0.6118
FAM47E	4	77185293	rs11097313	A	G	-0.0072	0.0144	0.614
FAM47E	4	77178827	rs79951067	T	C	-0.0488	0.0969	0.6143
FAM47E	4	77148271	rs143670970	C	G	0.049	0.0974	0.6146
FAM47E	4	77158854	rs2197102	T	G	0.009	0.018	0.6152
FAM47E	4	77156297	rs71607345	C	G	-0.0119	0.0237	0.6154
FAM47E	4	77179652	rs74806446	A	C	0.0487	0.0969	0.6156
FAM47E	4	77186447	rs141746656	A	G	-0.0358	0.0723	0.6203
FAM47E	4	77159039	rs3919711	A	C	-0.0088	0.018	0.6239
FAM47E	4	77182726	rs2869866	A	G	-0.0072	0.0146	0.6245
FAM47E	4	77159881	rs1441919	A	G	0.0088	0.018	0.6265
FAM47E	4	77161712	rs11737624	T	G	-0.0087	0.018	0.6303
FAM47E	4	77158640	rs2218264	A	G	-0.0086	0.018	0.6342
FAM47E	4	77173476	rs56371028	T	C	-0.0101	0.0212	0.6345
FAM47E	4	77181702	rs116501084	A	G	0.0461	0.0969	0.6345
FAM47E	4	77177027	rs17001726	A	G	-0.0119	0.0252	0.6349
FAM47E	4	77157947	rs35851125	A	G	0.0085	0.018	0.6379
FAM47E	4	77159712	rs1441921	T	C	-0.0085	0.018	0.6385
FAM47E	4	77181323	rs6813980	A	G	0.0068	0.0146	0.6407
FAM47E	4	77181437	rs6814195	A	G	0.0068	0.0146	0.6412
FAM47E	4	77180940	rs2119732	A	C	-0.0068	0.0146	0.6414
FAM47E	4	77144682	rs2034004	T	C	0.0068	0.0147	0.6417
FAM47E	4	77169256	rs6837841	A	G	-0.0084	0.018	0.6418
FAM47E	4	77153735	rs11097296	C	G	-0.0098	0.0213	0.6441
FAM47E	4	77166011	rs4859646	T	C	-0.0111	0.024	0.6441
FAM47E	4	77168813	rs113242109	A	T	0.0528	0.1145	0.6443
FAM47E	4	77161632	rs56896076	C	G	-0.011	0.024	0.647
FAM47E	4	77159717	rs1441920	A	G	-0.011	0.024	0.6478
FAM47E	4	77163426	rs12643198	A	G	0.0109	0.024	0.6503
FAM47E	4	77176852	rs74915725	A	G	-0.0441	0.0972	0.6503
FAM47E	4	77159700	rs1441922	A	G	-0.0082	0.018	0.6505
FAM47E	4	77194473	rs768981	A	G	-0.007	0.0155	0.6514
FAM47E	4	77213567	rs142560103	A	G	0.0349	0.0782	0.6558
FAM47E	4	77168444	rs78586815	A	G	0.0107	0.0241	0.6567
FAM47E	4	77184029	rs76769906	C	G	0.0428	0.0969	0.659
FAM47E	4	77180612	rs2119731	A	G	0.0064	0.0146	0.6598
FAM47E	4	77210187	rs10028743	T	C	0.0077	0.0177	0.6652
FAM47E	4	77156327	rs28530675	T	C	0.0078	0.0183	0.6684
FAM47E	4	77162428	chr4:77162428:D	A	AAG	0.0314	0.0742	0.6724
FAM47E	4	77176768	rs7655536	T	C	-0.0077	0.0181	0.6724

FAM47E	4	77216591	rs113337646	A	G	-0.1676	0.4005	0.6756
FAM47E	4	77155240	rs12331583	C	G	0.0089	0.0213	0.6763
FAM47E	4	77179601	rs17235935	T	C	-0.0059	0.0141	0.6772
FAM47E	4	77209078	rs191122953	T	C	-0.0651	0.1565	0.6777
FAM47E	4	77182012	rs62300777	T	C	-0.0058	0.0141	0.6812
FAM47E	4	77150930	rs116557578	A	G	0.0204	0.0497	0.6816
FAM47E	4	77174095	rs4859437	A	T	0.0099	0.0242	0.6831
FAM47E	4	77177846	rs6835986	A	G	0.0057	0.0141	0.6855
FAM47E	4	77172100	rs76836387	T	C	0.0103	0.0259	0.6909
FAM47E	4	77190347	rs116133387	A	G	-0.038	0.0969	0.695
FAM47E	4	77154759	chr4:77154759:D	A	ATG	0.0093	0.0237	0.6961
FAM47E	4	77187032	rs56060342	T	C	-0.0057	0.0147	0.6962
FAM47E	4	77198642	rs149808717	C	G	0.022	0.0566	0.6978
FAM47E	4	77174956	rs114597706	C	G	0.0372	0.0963	0.6994
FAM47E	4	77204570	rs78586494	T	C	-0.0379	0.0984	0.7001
FAM47E	4	77175031	rs115982466	A	G	-0.0371	0.0963	0.7002
FAM47E	4	77175134	rs116788490	T	G	-0.0369	0.0963	0.7017
FAM47E	4	77175050	rs114372500	A	G	-0.0368	0.0963	0.7027
FAM47E	4	77175263	rs116650795	A	G	0.0367	0.0963	0.7029
FAM47E	4	77184395	rs7677256	C	G	0.0056	0.0147	0.7046
FAM47E	4	77175542	rs114645222	A	G	0.0363	0.0962	0.7061
FAM47E	4	77175113	rs116394362	A	G	-0.0361	0.0963	0.7075
FAM47E	4	77154771	rs36031249	A	G	-0.0088	0.0236	0.7084
FAM47E	4	77198756	rs6532316	A	G	-0.0079	0.0213	0.7094
FAM47E	4	77186924	rs58804635	A	C	-0.0055	0.0147	0.7095
FAM47E	4	77199904	rs4859658	A	G	0.0064	0.0173	0.7101
FAM47E	4	77154709	rs35507310	T	G	0.0087	0.0236	0.7112
FAM47E	4	77154761	rs34369701	A	G	0.0087	0.0236	0.7125
FAM47E	4	77222206	rs149265677	C	G	-0.0407	0.1107	0.7131
FAM47E	4	77176090	rs75946022	C	G	0.0355	0.0972	0.7149
FAM47E	4	77210084	rs140822363	T	C	-0.0115	0.0316	0.7151
FAM47E	4	77202404	rs11097320	A	T	0.0063	0.0173	0.7152
FAM47E	4	77176187	rs115313834	A	G	-0.0353	0.097	0.7157
FAM47E	4	77174488	rs76523610	T	C	-0.0351	0.0964	0.716
FAM47E	4	77210435	rs111424216	T	G	0.0115	0.0316	0.716
FAM47E	4	77191112	rs2869872	T	C	0.0062	0.0173	0.7177
FAM47E	4	77193545	rs1441911	T	G	0.0061	0.0173	0.7219
FAM47E	4	77197537	rs28592037	A	T	-0.0075	0.0213	0.7231
FAM47E	4	77145002	chr4:77145002:D	CAGAGTG	C	-0.0484	0.1388	0.7275
FAM47E	4	77201487	rs1036788	T	C	-0.0059	0.0173	0.7315
FAM47E	4	77201332	rs1036787	A	G	0.0073	0.0213	0.732
FAM47E	4	77175778	rs116163997	T	C	-0.0325	0.0963	0.7355
FAM47E	4	77202762	rs1530295	A	G	0.0057	0.0173	0.7434
FAM47E	4	77201863	rs74883957	A	G	-0.0313	0.0971	0.7472
FAM47E	4	77186812	rs28492993	T	C	-0.0047	0.0147	0.7483
FAM47E	4	77201066	rs1441916	A	T	0.0055	0.0173	0.7513
FAM47E	4	77193047	rs77440835	A	G	0.0306	0.0968	0.7519
FAM47E	4	77171925	rs76485048	A	G	0.009	0.0288	0.7546
FAM47E	4	77187626	rs7687964	A	G	-0.0046	0.0147	0.7554
FAM47E	4	77153820	rs147993843	T	C	-0.049	0.1576	0.7557
FAM47E	4	77164337	rs10029710	T	C	-0.0058	0.0187	0.7564
FAM47E	4	77175245	rs12646113	T	C	-0.0052	0.017	0.7587
FAM47E	4	77191787	rs143128980	A	G	-0.0232	0.0756	0.7593
FAM47E	4	77173422	chr4:77173422:D	G	GC	0.0105	0.0344	0.761
FAM47E	4	77188244	rs4859652	A	G	0.0044	0.0147	0.7625

FAM47E	4	77202918	rs77046068	A	G	-0.0292	0.0971	0.7636
FAM47E	4	77193728	rs1441912	T	G	0.0052	0.0173	0.7655
FAM47E	4	77202713	rs4370157	A	G	0.0051	0.0173	0.7665
FAM47E	4	77165605	rs139128109	T	C	0.0083	0.0287	0.7727
FAM47E	4	77167713	rs1441906	T	C	0.0061	0.0213	0.7747
FAM47E	4	77167640	rs1348208	T	G	-0.006	0.0213	0.7772
FAM47E	4	77175108	rs7695543	A	C	-0.0047	0.0167	0.7776
FAM47E	4	77155591	rs6855568	T	C	-0.0044	0.0159	0.7821
FAM47E	4	77167242	rs1348206	A	G	-0.0058	0.0212	0.7831
FAM47E	4	77163276	rs10446680	T	C	0.0058	0.0213	0.7833
FAM47E	4	77171559	rs149881128	T	C	0.0501	0.1868	0.7885
FAM47E	4	77138635	rs193290840	T	C	-0.0522	0.1968	0.791
FAM47E	4	77168321	rs12499643	A	T	-0.0074	0.0288	0.7964
FAM47E	4	77188613	rs4859653	T	C	-0.0038	0.0147	0.7971
FAM47E	4	77211388	rs141387889	A	T	-0.0225	0.0878	0.7979
FAM47E	4	77158529	chr4:77158529:D	T	TG	0.0054	0.021	0.7983
FAM47E	4	77168112	rs12498798	T	C	0.0073	0.0288	0.7989
FAM47E	4	77169303	rs138275602	A	G	-0.0101	0.0399	0.7991
FAM47E	4	77203041	rs10015972	A	G	-0.005	0.0199	0.8
FAM47E	4	77203117	rs10016061	C	G	-0.005	0.0199	0.8003
FAM47E	4	77211864	rs56357209	T	C	0.0948	0.3955	0.8105
FAM47E	4	77176674	rs12645290	A	G	0.0082	0.0354	0.8175
FAM47E	4	77181755	rs2869863	A	G	-0.0056	0.0242	0.8176
FAM47E	4	77147980	rs2136053	A	G	-0.0301	0.1312	0.8186
FAM47E	4	77181939	rs2869864	A	G	-0.0055	0.0243	0.8191
FAM47E	4	77178379	rs6819794	A	G	-0.0032	0.0144	0.8223
FAM47E	4	77182273	rs13137733	T	C	0.0054	0.0243	0.8229
FAM47E	4	77162518	rs188892777	A	G	-0.0258	0.1158	0.8235
FAM47E	4	77174662	rs4859648	A	G	-0.0052	0.0235	0.8243
FAM47E	4	77174469	rs10856883	A	C	-0.0037	0.017	0.8253
FAM47E	4	77154421	rs36121867	T	C	-0.0048	0.0216	0.8255
FAM47E	4	77210630	rs139008362	A	G	0.0229	0.1044	0.8264
FAM47E	4	77224389	rs2174920	A	T	-0.004	0.0183	0.8275
FAM47E	4	77227434	rs182940983	A	G	-0.0237	0.1089	0.8281
FAM47E	4	77212211	rs77266888	A	C	-0.0087	0.0399	0.8282
FAM47E	4	77168749	chr4:77168749:D	CTT	C	-0.0056	0.0258	0.8298
FAM47E	4	77154865	rs11097297	T	C	-0.0034	0.0158	0.8304
FAM47E	4	77173739	rs75132248	T	C	0.0061	0.0287	0.8311
FAM47E	4	77181712	chr4:77181712:D	T	TAACTC	0.0052	0.0243	0.8311
FAM47E	4	77182409	rs34638846	A	G	-0.0051	0.0243	0.8329
FAM47E	4	77163810	rs112752720	T	C	-0.0191	0.0917	0.8351
FAM47E	4	77147795	rs2136054	A	G	-0.0272	0.1313	0.8362
FAM47E	4	77146405	rs2119730	T	C	-0.0035	0.0172	0.839
FAM47E	4	77203238	rs139764164	A	G	-0.0193	0.0966	0.8415
FAM47E	4	77172445	rs77457533	T	C	-0.0057	0.0287	0.8431
FAM47E	4	77153771	rs10222966	A	G	-0.0049	0.0255	0.847
FAM47E	4	77147513	rs10004443	A	G	-0.0039	0.0209	0.8507
FAM47E	4	77147679	rs2174922	A	G	0.0247	0.1315	0.8508
FAM47E	4	77218953	rs78884349	A	G	-0.0075	0.0403	0.8514
FAM47E	4	77147191	rs7672858	A	G	-0.0244	0.1315	0.8526
FAM47E	4	77151938	rs6846983	T	C	0.0032	0.0178	0.8582
FAM47E	4	77152235	rs6817492	A	C	-0.0032	0.0179	0.8585
FAM47E	4	77152706	rs4859434	T	C	-0.0032	0.0179	0.8585
FAM47E	4	77144833	rs72858510	A	G	-0.0249	0.1445	0.8632
FAM47E	4	77151490	rs1596117	T	C	-0.0031	0.0178	0.8633

FAM47E	4	77151084	rs10222765	T	C	0.0044	0.0255	0.8635
FAM47E	4	77151203	rs4859635	T	C	0.003	0.0178	0.8659
FAM47E	4	77179898	rs13122345	T	C	0.004	0.0242	0.8693
FAM47E	4	77150723	rs1948997	T	C	-0.0029	0.0178	0.8701
FAM47E	4	77180145	rs71607346	T	C	-0.0039	0.0242	0.8702
FAM47E	4	77153136	rs4859639	T	C	-0.0035	0.0219	0.8727
FAM47E	4	77163764	rs78756273	A	C	0.0041	0.0258	0.8748
FAM47E	4	77149645	rs1837868	A	G	-0.0029	0.0185	0.8757
FAM47E	4	77219750	rs80340869	T	C	0.0626	0.4066	0.8775
FAM47E	4	77154432	chr4:77154432:D	CA	C	0.0033	0.0212	0.8779
FAM47E	4	77149576	rs1837867	A	G	0.0027	0.0179	0.8788
FAM47E	4	77158218	rs113812080	T	G	-0.0209	0.1394	0.8807
FAM47E	4	77173914	rs13117238	A	C	0.0028	0.0193	0.8836
FAM47E	4	77153919	rs1583943	A	G	0.0031	0.0212	0.8854
FAM47E	4	77154193	rs34385906	T	C	-0.003	0.0212	0.8859
FAM47E	4	77154196	rs35472515	A	G	0.003	0.0212	0.8859
FAM47E	4	77179830	rs6851219	T	C	0.0034	0.0242	0.8885
FAM47E	4	77149335	rs4530661	A	C	-0.0022	0.0157	0.8893
FAM47E	4	77149119	rs12505005	T	C	-0.0042	0.03	0.8894
FAM47E	4	77176848	rs12643261	T	C	-0.0033	0.0243	0.8918
FAM47E	4	77175695	rs28636389	A	G	-0.0026	0.0197	0.8939
FAM47E	4	77138352	rs76591264	C	G	0.0101	0.0756	0.8941
FAM47E	4	77135787	rs116533523	T	G	-0.0057	0.0433	0.8945
FAM47E	4	77148055	rs2174921	C	G	0.0021	0.0157	0.8957
FAM47E	4	77161299	rs1596118	T	C	-0.0035	0.0285	0.901
FAM47E	4	77150296	rs1588214	T	C	0.0025	0.0211	0.9047
FAM47E	4	77186252	rs62300778	A	T	-0.0048	0.0399	0.9047
FAM47E	4	77152867	rs4859436	T	C	-0.0025	0.0211	0.9061
FAM47E	4	77153420	rs4859641	A	G	0.0025	0.0211	0.9061
FAM47E	4	77152547	rs4859432	T	C	-0.0025	0.0211	0.9062
FAM47E	4	77152539	rs4859431	C	G	-0.0025	0.0211	0.9064
FAM47E	4	77152052	rs4321663	A	G	0.0025	0.0211	0.9067
FAM47E	4	77150478	rs1828138	T	C	0.0025	0.0211	0.9074
FAM47E	4	77208196	rs6851326	T	G	-0.0021	0.018	0.9075
FAM47E	4	77151715	rs13136819	C	G	0.0024	0.0211	0.9084
FAM47E	4	77206172	rs188913211	A	C	0.0211	0.1842	0.9088
FAM47E	4	77151432	rs1596115	C	G	0.0024	0.0211	0.9092
FAM47E	4	77151017	rs34846125	A	C	0.0024	0.0211	0.9097
FAM47E	4	77156452	rs111815620	T	C	0.0142	0.1251	0.9099
FAM47E	4	77176897	rs12651582	T	C	0.0026	0.0235	0.9112
FAM47E	4	77150215	rs78908613	A	G	-0.0023	0.0211	0.9117
FAM47E	4	77150190	rs60225905	A	G	0.0023	0.0211	0.9123
FAM47E	4	77151474	rs1596116	T	C	-0.0023	0.0211	0.9125
FAM47E	4	77149099	rs4859430	A	G	-0.0023	0.0211	0.9138
FAM47E	4	77149945	chr4:77149945:I	CA	C	0.0024	0.0225	0.9142
FAM47E	4	77149742	rs1441903	A	C	0.0023	0.0211	0.9145
FAM47E	4	77148495	rs13114306	T	C	-0.0022	0.0211	0.9156
FAM47E	4	77148184	rs11531413	T	C	-0.0022	0.0211	0.9165
FAM47E	4	77146751	rs56275416	C	G	-0.0018	0.0172	0.9167
FAM47E	4	77154756	rs59293528	T	C	-0.0075	0.0714	0.9168
FAM47E	4	77174482	rs138745874	T	C	-0.0101	0.0974	0.9171
FAM47E	4	77148404	rs13140675	A	T	0.0022	0.0211	0.9183
FAM47E	4	77147073	rs13150767	A	G	-0.0021	0.0211	0.9202
FAM47E	4	77149948	rs11097291	T	C	0.0023	0.0225	0.9205
FAM47E	4	77155486	rs77332139	A	C	0.0028	0.0286	0.9209

FAM47E	4	77140464	rs188693863	A	G	0.008	0.0824	0.9227
FAM47E	4	77147300	rs9992683	T	C	-0.0024	0.0254	0.9253
FAM47E	4	77193228	rs72655583	T	C	-0.0024	0.0255	0.9254
FAM47E	4	77147265	chr4:77147265:I	CA	C	0.0016	0.0172	0.9268
FAM47E	4	77148899	rs9996456	T	C	0.0022	0.0256	0.93
FAM47E	4	77219405	rs183966465	A	G	0.0206	0.2369	0.9306
FAM47E	4	77222245	rs1532985	T	G	0.0088	0.1063	0.9342
FAM47E	4	77224649	rs7677358	C	G	0.0015	0.0183	0.935
FAM47E	4	77147969	rs4101061	A	G	0.0013	0.0157	0.9353
FAM47E	4	77225953	rs7694751	A	G	-0.0015	0.0183	0.9353
FAM47E	4	77146872	rs56277851	A	G	0.0014	0.0172	0.9365
FAM47E	4	77149947	rs11561677	A	T	0.0017	0.0226	0.9396
FAM47E	4	77193057	rs183715787	T	G	-0.0068	0.0922	0.9412
FAM47E	4	77155139	rs75868917	T	C	0.0021	0.0287	0.9418
FAM47E	4	77151300	rs79580103	A	G	0.0019	0.0287	0.9463
FAM47E	4	77225431	rs13150850	A	T	0.0012	0.0183	0.9479
FAM47E	4	77146871	rs55777203	T	C	-0.0011	0.0172	0.948
FAM47E	4	77146678	rs112796958	A	G	-0.0093	0.1444	0.9487
FAM47E	4	77151110	rs77514904	A	G	-0.0018	0.0287	0.9489
FAM47E	4	77151109	rs115105683	T	C	0.0018	0.0287	0.9491
FAM47E	4	77145441	rs12504181	A	G	0.0016	0.0256	0.9496
FAM47E	4	77151880	rs1441904	A	G	0.001	0.0157	0.9512
FAM47E	4	77152816	rs4859435	T	C	-0.001	0.0157	0.9513
FAM47E	4	77150468	rs75167214	A	G	0.0017	0.0287	0.9516
FAM47E	4	77177415	rs61740422	T	C	-0.0021	0.0355	0.953
FAM47E	4	77154244	chr4:77154244:D	A	AC	-0.0017	0.0287	0.9535
FAM47E	4	77154957	rs76504716	T	G	0.0017	0.0287	0.9538
FAM47E	4	77150655	rs76971852	T	C	-0.0016	0.0287	0.9549
FAM47E	4	77150838	rs12506229	A	G	0.0015	0.0287	0.958
FAM47E	4	77149868	chr4:77149868:D	CCTG	C	0.0015	0.0287	0.9596
FAM47E	4	77210033	rs144061236	T	C	-0.0029	0.0589	0.9606
FAM47E	4	77210195	rs7659748	C	G	-0.0009	0.018	0.9609
FAM47E	4	77157718	rs78252437	C	G	0.0012	0.0257	0.9617
FAM47E	4	77141957	rs56155931	A	G	0.0202	0.4285	0.9623
FAM47E	4	77225313	rs13104573	T	C	0.0008	0.0183	0.9652
FAM47E	4	77149772	rs78799603	T	G	-0.0012	0.0287	0.9671
FAM47E	4	77149540	rs113789302	A	G	-0.0011	0.0287	0.9699
FAM47E	4	77167499	rs1348207	A	T	-0.0006	0.0146	0.97
FAM47E	4	77148761	rs12504041	A	G	0.001	0.0287	0.9714
FAM47E	4	77152247	chr4:77152247:D	A	AAACCGTAC	-0.0049	0.1441	0.9728
FAM47E	4	77148601	rs113367757	T	C	0.001	0.0287	0.9732
FAM47E	4	77151630	rs79343926	T	G	0.0047	0.1443	0.9739
FAM47E	4	77211746	rs146457959	T	C	-0.0031	0.1072	0.9766
FAM47E	4	77183301	rs79408435	A	G	-0.0022	0.0896	0.9801
FAM47E	4	77142852	rs7659478	A	C	-0.0104	0.4281	0.9807
FAM47E	4	77175835	rs139482346	T	C	-0.0022	0.0926	0.9814
FAM47E	4	77148213	rs140146655	A	C	-0.0006	0.0287	0.983
FAM47E	4	77177353	rs61740367	A	G	0.0014	0.0813	0.9861
FAM47E	4	77211521	rs150769850	T	C	-0.0016	0.1071	0.9884
FAM47E	4	77224459	rs62300831	T	C	0.0012	0.0981	0.9902
FAM47E	4	77145892	rs76051648	T	C	-0.0003	0.0288	0.9911
FAM47E	4	77164792	rs62300773	T	G	-0.0001	0.0146	0.9964
OR2B2	6	27892729	rs79834417	A	C	-0.1952	0.0591	0.0009478
OR2B2	6	27892734	rs76041878	C	G	-0.1952	0.0591	0.0009481
OR2B2	6	27901575	chr6:27901575:D	CA	C	-0.1945	0.0591	0.0009946

OR2B2	6	27883269	rs1497525	A	C	0.1153	0.0353	0.001105
OR2B2	6	27898512	rs73394715	A	G	0.1046	0.0326	0.00133
OR2B2	6	27883201	rs1497526	A	G	0.1121	0.0352	0.001431
OR2B2	6	27893532	rs112798683	A	G	0.1066	0.0394	0.006785
OR2B2	6	27901041	rs7760871	A	G	0.1059	0.0394	0.007105
OR2B2	6	27880619	rs111280498	T	C	0.0935	0.0358	0.008958
OR2B2	6	27873596	rs73392694	A	G	0.0926	0.0361	0.01026
OR2B2	6	27881406	rs9885649	T	C	0.0917	0.0358	0.01044
OR2B2	6	27894732	chr6:27894732:D	A	AGT	0.0828	0.0324	0.01071
OR2B2	6	27887442	rs9468255	C	G	0.0898	0.0355	0.01144
OR2B2	6	27889527	rs9461420	A	C	-0.0993	0.0398	0.01265
OR2B2	6	27884916	rs9468254	A	G	0.0981	0.0398	0.01384
OR2B2	6	27904608	rs142559309	T	C	0.1581	0.0667	0.01779
OR2B2	6	27903316	chr6:27903316:D	T	TAAAGAG	1.7418	0.7497	0.02016
OR2B2	6	27896148	rs140383952	T	C	-0.0517	0.0228	0.0233
OR2B2	6	27870772	rs9468252	A	G	-0.0834	0.0369	0.02361
OR2B2	6	27905181	rs190770443	A	C	0.573	0.2656	0.03101
OR2B2	6	27905185	rs182964030	A	G	-0.573	0.2656	0.03101
OR2B2	6	27902589	rs138888559	A	G	-0.5725	0.2657	0.03116
OR2B2	6	27895399	rs146559954	T	C	0.5715	0.2657	0.03149
OR2B2	6	27898706	rs75269802	A	G	0.1657	0.0839	0.04836
OR2B2	6	27902087	rs77739671	A	C	0.1643	0.0839	0.05021
OR2B2	6	27891211	rs112080944	A	G	-0.1641	0.0839	0.05054
OR2B2	6	27893589	rs111867038	A	G	0.1635	0.0839	0.05134
OR2B2	6	27898030	rs115846780	T	C	0.1634	0.0839	0.05149
OR2B2	6	27876070	rs111737144	T	C	-0.1642	0.0844	0.0516
OR2B2	6	27895266	rs76147830	A	G	0.1633	0.0839	0.0517
OR2B2	6	27876707	rs112991750	A	G	-0.1635	0.0844	0.05268
OR2B2	6	27872472	rs76154481	T	C	0.163	0.0845	0.05364
OR2B2	6	27886830	rs2130357	T	C	0.0283	0.0164	0.08491
OR2B2	6	27896799	rs71559054	A	C	0.0459	0.0287	0.1097
OR2B2	6	27905509	rs67040724	T	C	0.0455	0.0287	0.1124
OR2B2	6	27879982	rs61742093	A	G	0.0452	0.0287	0.1157
OR2B2	6	27879200	rs34788973	A	C	-0.0451	0.0287	0.1161
OR2B2	6	27878846	rs189146064	T	C	-0.3737	0.2392	0.1182
OR2B2	6	27885793	rs188215669	T	C	0.1889	0.1263	0.1347
OR2B2	6	27870817	rs62401414	A	G	-0.0904	0.063	0.1513
OR2B2	6	27900709	chr6:27900709:D	CTT	C	-0.1362	0.0967	0.1589
OR2B2	6	27904019	rs9461421	T	G	-0.1298	0.097	0.1812
OR2B2	6	27895645	rs9468257	T	G	0.1296	0.0971	0.1818
OR2B2	6	27886251	rs73392701	T	C	-0.1286	0.0972	0.1859
OR2B2	6	27881809	rs147945313	T	C	-0.4386	0.3475	0.2069
OR2B2	6	27878738	rs149878	T	G	-0.021	0.0167	0.207
OR2B2	6	27871553	rs9468253	C	G	-0.121	0.0982	0.2179
OR2B2	6	27894196	rs11966528	T	G	-0.1175	0.0965	0.2236
OR2B2	6	27883968	rs184853407	A	G	0.4105	0.34	0.2273
OR2B2	6	27905916	rs149392785	T	C	0.0989	0.082	0.2276
OR2B2	6	27884012	rs13218875	T	C	-0.0337	0.0279	0.2281
OR2B2	6	27887237	rs77619023	A	G	0.1223	0.1036	0.2379
OR2B2	6	27896386	rs185708448	T	C	0.1656	0.1513	0.2737
OR2B2	6	27877985	rs149855	A	G	-0.0195	0.0178	0.2739
OR2B2	6	27871993	rs78498127	A	G	0.0406	0.0377	0.2813
OR2B2	6	27903467	rs10456357	A	G	-0.0396	0.0373	0.2884
OR2B2	6	27895213	rs156737	A	G	-0.0159	0.0152	0.2964
OR2B2	6	27892913	rs114951800	T	G	0.0774	0.0749	0.3015

OR2B2	6	27904077	rs9461422	T	C	-0.0973	0.0954	0.308
OR2B2	6	27885972	rs142484160	A	G	0.069	0.0693	0.3192
OR2B2	6	27881721	rs75249060	C	G	-0.0736	0.0785	0.3483
OR2B2	6	27872486	rs79967824	T	C	0.0427	0.0456	0.3487
OR2B2	6	27902869	rs7742529	A	G	0.0351	0.0375	0.35
OR2B2	6	27897620	rs143428462	A	G	-0.1174	0.1264	0.3529
OR2B2	6	27872759	rs9380028	A	C	0.0418	0.0456	0.3595
OR2B2	6	27879551	rs9368537	C	G	-0.041	0.0452	0.3647
OR2B2	6	27904255	rs147818758	T	C	-0.1445	0.1619	0.3718
OR2B2	6	27888255	rs112274365	T	C	-0.3355	0.3915	0.3914
OR2B2	6	27886693	rs80201717	T	C	0.1337	0.1611	0.4066
OR2B2	6	27876479	rs113757670	A	T	-0.2621	0.3212	0.4145
OR2B2	6	27880569	rs185727584	C	G	-0.0533	0.0653	0.4145
OR2B2	6	27876311	rs113591880	A	G	0.3687	0.4545	0.4172
OR2B2	6	27887275	rs1015075	A	G	0.0363	0.0451	0.4208
OR2B2	6	27882895	rs192415638	T	C	0.0992	0.1238	0.4231
OR2B2	6	27901061	rs9468259	A	G	-0.0795	0.0995	0.4246
OR2B2	6	27874523	rs113140430	T	C	0.3596	0.454	0.4284
OR2B2	6	27873461	rs113325551	A	G	-0.3589	0.4546	0.4298
OR2B2	6	27870770	rs183949249	A	G	0.3477	0.4544	0.4442
OR2B2	6	27878268	rs72848752	A	C	-0.0219	0.0289	0.4484
OR2B2	6	27894803	rs111997627	A	G	-0.063	0.084	0.4529
OR2B2	6	27886674	rs113565609	A	G	-0.3601	0.4804	0.4535
OR2B2	6	27895082	rs9380029	T	G	-0.0332	0.045	0.4611
OR2B2	6	27891993	rs11962485	T	C	0.0723	0.0992	0.4662
OR2B2	6	27897982	rs150891093	T	G	-0.2053	0.3018	0.4963
OR2B2	6	27904345	rs76106973	A	G	-0.0526	0.0778	0.4986
OR2B2	6	27886437	rs114833655	T	C	-0.3085	0.4575	0.5001
OR2B2	6	27877446	rs188015	A	T	-0.0159	0.0238	0.5039
OR2B2	6	27895913	rs114766838	T	C	-0.1695	0.2547	0.5057
OR2B2	6	27873931	rs72847381	A	G	0.0187	0.0284	0.5105
OR2B2	6	27875823	rs73392695	A	G	0.0656	0.1	0.5119
OR2B2	6	27901391	chr6:27901391:DCTAGAACCT		C	-0.0135	0.0211	0.5231
OR2B2	6	27904952	rs16868158	T	C	0.0532	0.0859	0.5357
OR2B2	6	27894889	rs112801350	T	G	-0.0532	0.086	0.5363
OR2B2	6	27897078	rs111289065	A	C	-0.0532	0.086	0.5363
OR2B2	6	27899221	rs75963251	A	T	0.0532	0.086	0.5363
OR2B2	6	27901314	rs149018156	T	G	0.0535	0.0876	0.5411
OR2B2	6	27902980	rs150835063	T	C	0.0535	0.0876	0.5411
OR2B2	6	27904636	rs150947515	T	C	0.0535	0.0875	0.5412
OR2B2	6	27901078	rs137904627	A	G	0.0535	0.0876	0.5413
OR2B2	6	27905591	rs112438225	A	G	0.0535	0.0875	0.5413
OR2B2	6	27893958	rs117232314	A	G	0.0534	0.0876	0.5422
OR2B2	6	27870761	rs181075040	T	C	0.2403	0.4216	0.5686
OR2B2	6	27904841	rs114218732	T	G	-0.0746	0.1347	0.5797
OR2B2	6	27884447	rs145570903	A	G	0.0491	0.0895	0.5831
OR2B2	6	27902172	rs425159	A	T	-0.04	0.0759	0.5985
OR2B2	6	27891116	rs151280335	A	G	-0.0435	0.0877	0.6197
OR2B2	6	27890164	rs111817601	A	G	0.0435	0.0877	0.6198
OR2B2	6	27898508	rs111763699	A	G	0.0519	0.109	0.6344
OR2B2	6	27886387	rs145590357	A	C	0.0415	0.0879	0.637
OR2B2	6	27903238	rs111965601	T	C	0.0401	0.0852	0.638
OR2B2	6	27875697	rs200962	A	G	0.034	0.0731	0.6423
OR2B2	6	27875871	rs143789967	A	G	-0.2449	0.5452	0.6533
OR2B2	6	27895787	rs112168266	T	C	0.0377	0.0864	0.6625

OR2B2	6	27884864	rs149854	A	G	-0.031	0.0728	0.6706
OR2B2	6	27898541	rs77889226	T	C	-0.0363	0.0864	0.6744
OR2B2	6	27877868	rs149226	A	G	-0.0312	0.0743	0.6746
OR2B2	6	27895920	rs113893965	A	G	0.0362	0.0864	0.6749
OR2B2	6	27896523	rs150356835	T	G	0.0362	0.0864	0.6752
OR2B2	6	27874626	rs138821634	A	C	0.0587	0.1411	0.6772
OR2B2	6	27889285	rs6927093	A	C	-0.0353	0.0865	0.6832
OR2B2	6	27887716	rs149879	T	C	0.029	0.0729	0.6905
OR2B2	6	27885794	rs181790456	A	G	0.1368	0.3466	0.693
OR2B2	6	27891201	rs112051980	A	G	-0.0304	0.0852	0.7213
OR2B2	6	27872898	rs75165919	A	G	-0.0131	0.0372	0.7258
OR2B2	6	27881359	rs145970942	C	G	0.186	0.5358	0.7285
OR2B2	6	27898428	rs72848762	A	T	-0.0257	0.0798	0.7469
OR2B2	6	27886001	rs181875225	T	C	-0.0605	0.1964	0.7582
OR2B2	6	27901419	rs183827352	A	G	-0.06	0.2021	0.7664
OR2B2	6	27903118	rs16868154	T	C	-0.0233	0.0856	0.7855
OR2B2	6	27877928	rs111574878	T	C	-0.0262	0.1026	0.7986
OR2B2	6	27878609	rs111639243	A	G	0.1098	0.431	0.7989
OR2B2	6	27890762	rs148772874	A	C	0.1343	0.5284	0.7993
OR2B2	6	27886051	rs6908688	A	G	-0.0063	0.0285	0.8254
OR2B2	6	27893730	chr6:27893730:D	A	AAG	0.0164	0.0744	0.8258
OR2B2	6	27884127	rs148298110	A	T	-0.0062	0.0286	0.8292
OR2B2	6	27884199	rs140414938	A	G	0.006	0.0286	0.8346
OR2B2	6	27897999	rs139687024	A	G	0.0137	0.0658	0.8347
OR2B2	6	27895000	rs79386517	T	C	-0.008	0.0386	0.8356
OR2B2	6	27902992	rs7742858	T	C	-0.0046	0.0238	0.8449
OR2B2	6	27884228	rs62401415	A	G	0.0063	0.0333	0.8507
OR2B2	6	27892159	rs189811866	C	G	0.0198	0.1055	0.8512
OR2B2	6	27893729	chr6:27893729:D	A	AAAG	0.0136	0.0745	0.8553
OR2B2	6	27877505	rs75916370	T	C	-0.035	0.1938	0.8567
OR2B2	6	27885356	rs731132	T	G	0.0026	0.015	0.8641
OR2B2	6	27897257	rs145321896	A	G	-0.0035	0.0237	0.8834
OR2B2	6	27886877	rs72848760	A	T	0.0044	0.0302	0.8854
OR2B2	6	27902488	rs72848763	A	G	-0.0044	0.0302	0.8854
OR2B2	6	27881147	rs12181629	A	T	-0.0079	0.0563	0.8879
OR2B2	6	27893166	rs34899043	T	C	-0.0037	0.0286	0.8965
OR2B2	6	27895504	rs9468256	A	G	0.0026	0.0205	0.8981
OR2B2	6	27877708	chr6:27877708:I	T	TC	-0.0038	0.0305	0.9007
OR2B2	6	27904181	rs6910968	T	C	-0.0028	0.0237	0.9058
OR2B2	6	27872832	rs200963	A	G	0.004	0.0398	0.9207
OR2B2	6	27885629	chr6:27885629:D	T	TGGA	-0.0057	0.0581	0.9216
OR2B2	6	27885630	chr6:27885630:D	G	GGAGT	-0.0057	0.0581	0.9217
OR2B2	6	27875641	rs72847383	A	G	0.0029	0.0303	0.9248
OR2B2	6	27903572	rs16868156	T	C	-0.0037	0.0399	0.9262
OR2B2	6	27873090	rs72847379	A	G	0.0028	0.0303	0.9274
OR2B2	6	27878269	rs72848753	C	G	-0.0025	0.0308	0.9341
OR2B2	6	27885422	rs192642430	T	C	0.0269	0.3622	0.9409
OR2B2	6	27885980	rs142863425	T	C	-0.0017	0.0242	0.9425
OR2B2	6	27877999	rs114354963	A	G	0.0038	0.0565	0.9458
OR2B2	6	27899687	rs9393864	A	G	0.002	0.0407	0.9605
OR2B2	6	27873460	rs2130355	T	C	-0.0014	0.0401	0.9729
OR2B2	6	27890631	rs6933825	A	G	-0.0006	0.0205	0.9767
OR2B2	6	27879397	rs34957169	T	C	-0.0009	0.0301	0.9773
OR2B2	6	27900354	rs9393865	T	G	0.0011	0.0406	0.9784
OR2B2	6	27873454	rs2130356	A	T	0.0009	0.0409	0.9831

OR2B2	6	27878553	rs72848755	T	C	-0.0006	0.0302	0.9835
OR2B2	6	27878458	rs72848754	A	T	0.0006	0.0302	0.9848
OR2B2	6	27885437	rs9357053	A	G	-0.0006	0.0407	0.9878

Abbreviations: EA, effect allele; NEA, non-effect allele
